# Surgical Outcomes in Chiari 1 and Chiari 1.5 Malformation Treated by Posterior Fossa Reconstruction: A Comprehensive Analysis of 110 Pediatric Cases and Literature Review

**DOI:** 10.3390/jcm13133852

**Published:** 2024-06-30

**Authors:** Maria A. Poca, Diego Lopez-Bermeo, Dulce Moncho, Alex Ferre, Angel Sanchez-Montañez, Olga Mestres, Sandra Galve, Juan Sahuquillo

**Affiliations:** 1 Department of Neurosurgery, Vall d’Hebron University Hospital, Passeig Vall d’Hebron 119-129, 08035 Barcelona, Spain; diegofernando.lopez@vallhebron.cat (D.L.-B.); sahuquillo@neurotrauma.net (J.S.); 2 Neurotraumatology and Neurosurgery Research Unit, Vall d’Hebron Institut de Recerca (VHIR), Vall d’Hebron Hospital Universitari, Vall d’Hebron Barcelona Hospital Campus, Passeig Vall d’Hebron 119-129, 08035 Barcelona, Spain; dulcemaria.moncho@vallhebron.cat (D.M.); alejandro.ferre@vallhebron.cat (A.F.); olga.mestres@vallhebron.cat (O.M.); 3 Department of Surgery (Neurosurgery), Universitat Autònoma de Barcelona, 08193 Bellaterra, Spain; 4 Clinical Neurophysiology Department, Vall d’Hebron Hospital Universitari, Vall d’Hebron Barcelona Hospital Campus, Passeig Vall d’Hebron 119-129, 08035 Barcelona, Spain; 5 Sleep Unit, Pneumology Department, Vall d’Hebron Hospital Universitari, Vall d’Hebron Barcelona Hospital Campus, Passeig Vall d’Hebron 119-129, 08035 Barcelona, Spain; 6 Department of Pediatric Neuroradiology, Institut de Diagnostic per la Imatge (IDI), Vall d’Hebron Hospital Universitari, Vall d’Hebron Barcelona Hospital Campus, Passeig Vall d’Hebron 119-129, 08035 Barcelona, Spain; angel.sanchez.idi@gencat.cat; 7 Pediatric Anesthesiology Department, Vall d’Hebron Hospital Universitari, Vall d’Hebron Barcelona Hospital Campus, Passeig Vall d’Hebron 119-129, 08035 Barcelona, Spain; sandra.galve@vallhebron.cat

**Keywords:** Chiari malformation, Chiari malformation type 1, Chiari malformation type 1.5, children, outcome, posterior fossa reconstruction, syringomyelia, treatment

## Abstract

**Background/Objectives:** The management of Chiari malformations (CMs) remains a clinical challenge and a topic of great controversy. Results may vary between children and adults. The purpose of the current single-center study is to critically assess the one-year surgical outcomes of a cohort of 110 children with CM-1 or CM-1.5 who were treated using “posterior fossa reconstruction” (PFR), a surgical technique described in 1994 that has since been used in both adults and children. We also review the literature and discuss the possible causes of the drawbacks and pitfalls in children in whom PFR was ineffective in controlling the disease. **Methods:** The present cohort was selected from a prospective registry of adults and children with CMs collected since 2006. Patients included in this study were selected from a group of children with CMs who were operated on in our Pediatric Neurosurgical Unit between 1 January 2007 and 31 November 2023. Surgical outcome was defined based on clinical and neuroradiological results as very good, good, or bad. **Results:** The mean age of our child cohort was 9.9 ± 4.7 years, with 54 girls (49%) and 56 boys (51%). Sixty-six children had CM-1 (60%) while forty-four had CM-1.5 (40%). Following surgery, there was no neurological worsening or death among the children. Most children (70%) had an uneventful recovery and were discharged home on average one week after surgery. However, in 33 children (30%), we recorded at least one postoperative adverse event. Aseptic meningitis syndrome was the most frequent adverse event (n = 25, 22.7%). The final surgical outcome was evaluated one year after PFR by using both clinical and neuroradiological results. The one-year surgical outcome was excellent in 101 children (91.9%), good in 5 (4.5%), and bad in 4 (3.6%). **Conclusions:** PFR significantly enlarges the volume of the posterior fossa and recreates a CSF environment that generates buoyancy of the cerebellum, with a high percentage of excellent and good clinical results evaluated one year post-surgery.

## 1. Introduction

Chiari malformations (CMs) were originally described in 1891 by the Austrian pathologist Hans Chiari from the autopsy of children. Chiari classified these deformities into three separate types, namely CM-1, CM-2, and CM-3 [1]. In 1895, he added a new phenotype (CM-4) [2]. Over the past twenty years, additional subcategories have been introduced, including CM-0, CM-0.5, CM-1.5, CM-3.5, and CM-5, bringing the total count of described variants to nine [3]. For readers seeking a detailed historical analysis of the classification, we recommend consulting recently published reviews [3,4].

The pathological hallmark of CMs is the displacement of cerebellar tissue through the FM, leading to compression and distortion of adjacent neural structures [5]. The clinical manifestations can vary widely, encompassing headaches, dizziness, coordination difficulties, and even more severe neurological deficits when patients have an associated syringomyelia (Syr). Chiari malformation type 1 (CM-1) is the phenotype most frequently found both in adults and children referred to neurosurgery consultation. In CM-1 and Chiari malformation type 1.5 (CM-1.5), the frequency of Syr in patients who underwent surgery ranges from 30 to 75% [6,7,8,9,10].

There has been enormous advancement in comprehending the development and genetic transmission linked to CMs; however, the treatment of these conditions still creates clinical challenges and considerable disagreement among clinicians. The ongoing debate centers around strategies for managing children who show no symptoms but have Syr and for determining the most effective surgical method for treating patients with CM-1 once surgery has been decided. Currently, the only treatment for reducing symptoms and control associated Syr in CMs is posterior fossa decompression (PFD) surgery, with or without opening of the dura mater and/or arachnoid, with or without an expansive duraplasty, and with or without coagulation/excision of the cerebellar tonsils [11]. The wide variety and heterogeneity of the indications for surgery have generated some recent consensus conferences, systematic reviews, and evidence-based guidelines promoted by CM patient associations and specialists, both in the EU and the USA [11,12,13,14].

In the most recent guidelines endorsed by the American Association of Neurological Surgeons (AANS), the Congress of Neurological Surgeons (CNS), and the Bobby Jones Chiari and Syringomyelia Foundation, the recommendation for symptomatic CM-1 patients was that “*…either posterior fossa decompression or posterior fossa decompression with duraplasty may be utilized as a first-line treatment to improve preoperative symptoms*” [14]. However, despite the large number of studies reviewed, the strength of this recommendation was only Grade C based on Class III evidence [14]. In part, the difficulties in obtaining good quality evidence rest on the fact that, once the decision to operate has been made, there is disagreement among neurosurgeons on what should be the standard surgical technique for PFD. These discrepancies make PFD a technique with excessive variability between institutions and neurosurgeons, and it has evolved into a non-standard operation with numerous variants. This makes comparing series unreliable and assessing surgical outcomes challenging. The extent of occipital bone removal in PFD ranges from a minimal foramen magnum decompression with or without removal of the posterior arch of the atlas to a more standardized 3 × 3–4 cm craniectomy, or even very extensive bone removal [10,15].

Many neurosurgeons have underscored the importance of performing a small bone decompression to prevent a ‘slump’ of the hindbrain into the craniectomy, as described by Duddy and Williams [16]. Once the craniectomy is completed, neurosurgeons use different strategies: some perform bone decompression without opening the dura, others make an incision or delamination of the outer layer of the dura mater, while others opt for dural opening without graft placement, or with the utilization of various grafts (autografts, allografts, or xenografts) [10,17,18]. In the recent International Consensus Document (ICD) for adults, panelists reached a 100% agreement to recommend PFD with duraplasty in patients with CM-1 and Syr [12]. However, the agreement was reduced to 75% when Syr was not present because of the risks of duraplasty mainly related to cerebrospinal fluid (CSF) leaks [12]. Regarding duraplasty, 81.3% of the experts rejected artificial grafts in favor of autologous grafts or allografts [12], with a high agreement (93.8%) on conducting a waterproof closure of the duraplasty with non-resorbable stitches [12]. However, in the ICD for children, 18.2% of the members disagreed with the PFD and duraplasty approach and 80.9% of them considered PFD without any intradural manipulation as an alternative for children with symptomatic CM1 without Syr if the “…family accepts the perspective of possible second surgery” [13].

CM pathogenesis has been clarified since the early 1990s, and today it is widely accepted that CM-1 occurs during human embryogenesis [3]. Experimental models using vitamin A or trypan blue as teratogens in rodents, the Chiari-like abnormalities found in some dog breeds (i.e., King Charles Cavalier Spaniels), and neuroimaging studies in patients with CMs indicated that the main factor in CM-1 pathogenesis is an underdeveloped skull base and small PF [19,20]. In 1981, Marín-Padilla hypothesized that the origin of most axial skeletal dysraphic disorders, including CMs, was essentially a primary paraxial mesoderm insufficiency [21,22]. Clinical studies have supported this hypothesis, and research conducted on CM-1 and CM-2 patients has consistently found, in most cases, a small PF, short clivus, increase in the tentorial slope, and significant reduction in PF volume [8,23,24,25,26,27].

As remarked by Batzdorf [28], the main goals of surgical treatment in patients with CM-1 should be 1. to relieve the craniospinal pressure differential that exists above and below the subarachnoid block at the FM level by restoring the continuity of the subarachnoid space; 2. to eliminate the Syr when present; and 3. to relieve the brain stem compression [28]. In 1994, we proposed a surgical procedure for CM-1 that we called ‘posterior fossa reconstruction’ (PFR) [15,29], a term suggested by Marín-Padilla in his seminal study on experimentally induced CMs in which he stated that “*…reconstruction and enlargement of the posterior cerebral fossa space may be the only necessary thing to do in this malformation. The reconstruction and enlargement of the available space within the posterior cerebral fossa could provide the extra room needed for the normal growth of the cerebellum, thus avoiding any type of compression of the nervous structures within that region*” [22]. In a pilot study reported in 1994, we showed that a large suboccipital craniectomy permits a significant volumetric enlargement of the PF, a repositioning of the cerebellar tonsils, and the creation of an artificial cisterna magna, which acts as a buoyant upward force on the cerebellum to keep it afloat and alleviate FM crowding [15]. In our pilot study, ten patients treated with PFR were compared with the same number of historical controls (small suboccipital craniectomy). In the control group, we found a variable ‘slump’ of the cerebellum in all but one patient [15]; however, in all patients in the PFR group, a mean upward migration of the cerebellum—measured by the fastigium–basal line—of 6.2 mm was observed in all patients associated with a generous pseudocisterna magna in postoperative magnetic resonance imaging (MRI) [15]. Our data showed that a larger cisterna magna was protective of cerebellar ptosis, a finding in line with those of Duddy and Williams, who reported, via quantitative assessment of hindbrain migration, that the “slump was greater in patients with a small artificial cisterna magna and little CSF space below the cerebellum, and was less amongst those with a large bulging cisterna magna” [16]. In a comment on our paper, Batzdorf wrote “The theory that the enlarged cisterna magna provides an upward push to the cerebellum and brain stem, and that this is the critical aspect of surgical therapy, is fascinating and will require further testing” [30]. Since 1994, we have used this technique in 504 adults and children with CMs, with minor modifications incorporated for the surgical treatment of CM-1.5.

There is evidence suggesting that CM-1 outcomes may differ between pediatric and adult patients [31]. The present single-center study aims to critically evaluate a group of 110 children diagnosed with CM-1 and CM-1.5, who were treated at our institution using PFR. We discuss the changes incorporated into the original technique, its rationale, and the extended surgical approach used for patients with CM-1.5. We consider the challenges involved in the surgical management of this intricate disease in pediatric patients, including both the lessons learned and potential drawbacks and pitfalls in the children in whom PFR was not effective in controlling the disease [11].

## 2. Patients and Methods

Patients included in this study were selected from a group of children diagnosed with CMs who were referred to the Pediatric Neurosurgical Unit of the Department of Neurosurgery at Vall d’Hebron University Hospital (VHUH) between 1 January 2007 and 30 November 2023. VHUH is a tertiary care setting and a reference center for CMs and other congenital anomalies of the CVJ in adults and children. The present cohort was selected from a prospective registry of adults and children with CMs collected since 2006 (PROSAC: Prospective Registry of Patients with Chiari Malformations in Adults and Children) [3]. In 2013, all MRIs in PROSAC were reviewed for all patients included before that date and reclassified by two of the authors (JS and MAP) into the three phenotypes that are currently accepted: CM-0, CM-1, and CM-1.5 [12,13,32]. At the time of query, PROSAC contained 1164 patients registered between 1 December 2007 and 31 January 2024. This does not include patients with CM-2 and other, more extreme, abnormalities, such as CM-3 and CM-4.

**Figure 1:**
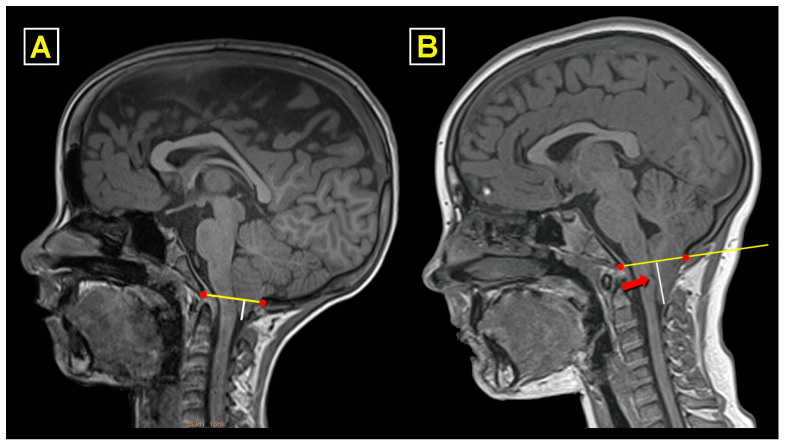
((**A**) (**left**)): Chiari malformation type 1, with a tonsillar herniation (TH) of 7.6 mm below the foramen magnum (McRae’s line in yellow) in midsagittal T1-weighted magnetic resonance imaging. ((**B**) (**right**)): Chiari malformation type 1.5. In this child, TH was 22 mm, and the patient had a caudal descent of the obex (red arrow) located 11 mm below McRae’s line.

Despite the recognized lack of reliability of tonsillar herniation (TH) as a single criterion for the diagnosis of CM-1 [33,34], to make our series comparable, we only selected patients with a diagnosis of classic CM-1 based on the Barkovich criterion (TH > 3 mm below the basion–opisthion or McRae’s line) in a midsagittal T1-weighted MRI [35]. To split our cohort between CM-1 and CM-1.5, we used the criteria of Tubbs et al. [36], and CM-1.5 was diagnosed when, in addition to TH > 3 mm, patients also had a caudal descent of the brainstem recognized by the obex located below McRae’s line (Figure 1) [36].

Inclusion criteria consisted of children aged 18 or younger with CM-1 or CM-1.5, no prior Chiari-related surgery, a minimum follow-up of 12 months, and at least one control MRI within this time frame to verify the adequacy of surgical decompression, tonsil ascent, and the reduction achieved in Syr extension and thickness. Patients were excluded if they presented complex CVJ malformations, defined as the coexistence of TH with at least two of the following abnormalities: significant retroflexed odontoid, basilar invagination (BI), platybasia, severe bone abnormalities in the C0–C2 complex, unilateral or bilateral occipital condyle hypoplasia, atlanto-occipital assimilation, and other abnormalities that cause anterior compression of the cervicomedullary junction [1,6,37]. Complex CVJ malformations were excluded because these patients require different clinical management and often need multiple surgical procedures, such as anterior approaches and/or occipitocervical fusions.

Within the study period (December 2007 to November 2023), 334 children (176 boys [52.7%] and 158 girls [47.3%]; mean age: 10.5 ± 4.5 years, min: 1, max: 18) with CMs were evaluated at the Pediatric Neurosurgical Unit of the VHUH. Of these 334 children, 34 (10.2%) were diagnosed with a complex malformation of the CVJ (CM-CVJ), 13 children had skeletal dysplasia without cerebellar ectopy, 17 were referred after being operated on at another center, 5 patients had CM-0, and 2 had CM-2. Of the remaining 263 children, 121 (46%) underwent surgical treatment. In 10 cases, surgical treatment was limited to the implantation of an epidural sensor for continuous intracranial pressure (ICP) monitoring (n = 2) or a ventriculoperitoneal shunt (n = 8). Pre- and postsurgical MRIs were lost in one patient. The present study focused on the remaining 110 patients with CM-1 or CM-1.5, in whom a PFR was performed.

### 2.1. Study Protocol and Neuroradiological Workup

**Figure 2:**
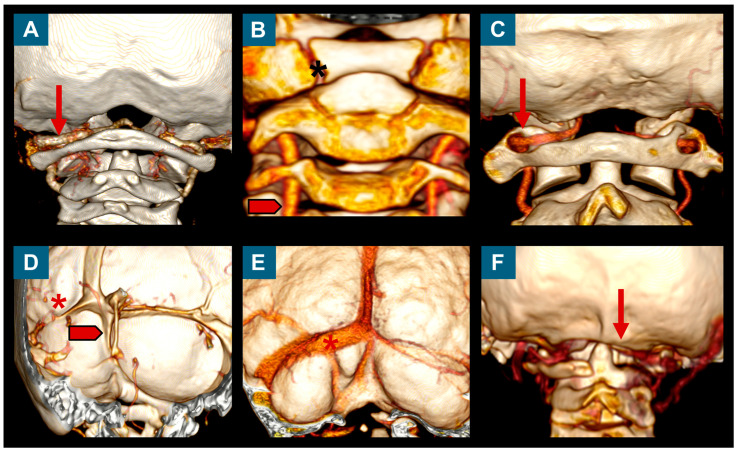
Examples of some relevant findings for planning posterior fossa surgery observed in the three-dimensional CT reconstructions. (**A**). Significant asymmetry and medialization of the left vertebral artery (V_3_) segment when crossing the atlas left vertebral artery sulcus (arrow). (**B**). Single-anterior arch ossification center and neurocentral synchondroses (asterisk) with a significant asymmetry of the vertebral arteries, with a predominancy of the right (arrowhead). (**C**). Asymmetrical *ponticulus posticus*, an infrequent anatomical variant that connects the superior articular fossa of the atlas with their posterior arch. (**D**). Significant hypoplasia of the right transverse sinus and a significant occipital sinus draining into the left transverse sinus (arrowhead). (**E**). Significant hypoplasia of the left transverse sinus with infrequent abnormalities and anastomosis in the venous pattern of the sinus around the foramen magnum that, if undetected before surgery, might make the surgery cumbersome due to copious venous bleeding when opening the dura mater. (**F**). Absence of the right posterior arch of the atlas or Currarino type B anomaly [39]. In this situation, the ipsilateral vertebral artery is unprotected and could be damaged during surgery.

The routine study protocol for these patients was the same as that described elsewhere for adults, which continues to be used today [7]. It includes clinical anamnesis, neurological examination, cranial and whole spinal MRI, and neurophysiological examination (brainstem auditory evoked potentials [BAEPs], somatosensory evoked potentials [SEPs], and a sleep study). In children requiring surgical treatment, computed tomography angiography of the CVJ is routinely conducted with coronal, sagittal, and 3D reconstructions to exclude any significant structural skeletal abnormalities of the CVJ (atlas assimilation, atlanto-occipital fusion, Klippel–Feil malformation, etc.) and abnormalities in the transverse sinus, and to rule out a malposition of vertebral arteries (Figure 2) [38]. In selected patients in whom CVJ instability is presumed, the pre-operative assessment includes flexion and extension X-ray studies of the CVJ.

**Figure 3:**
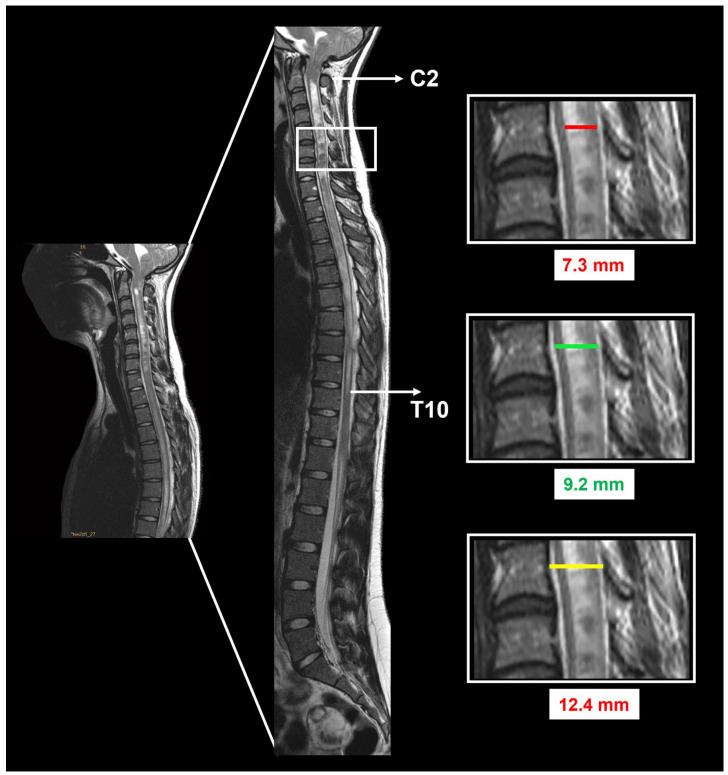
Calculation of the extension and diameter of the syrinx cavity. The extension of the syringomyelia (Syr) was quantified as the number of vertebrae between the superior and inferior limits of the cavity (in this patient, between C2 and T10). To calculate the syringo-cord ratio (SCR) and the syrinx–canal index (SCI), we measured the Syr maximum anteroposterior (AP) diameter at its widest level on the midsagittal MRI (red line, 7.3 mm), the maximum AP diameter of the spinal cord at the same level (green line, 9.2 mm), and the spinal canal diameter (yellow line, 12.4 mm) at the same level at which the maximum AP diameter of the syrinx was measured (white frame). The SCR was calculated by dividing the syrinx’s maximum AP distance by the spinal cord’s AP (SCR %: AP diameter of the syrinx/AP diameter of the spinal cord × 100 = 79.3%). The SCI was calculated by dividing the syrinx’s maximum AP distance by the spinal canal’s AP at the same level (SCI %: AP diameter of the syrinx/AP diameter of the spinal canal × 100 = 58.9%).

In addition to detecting TH below the FM, the presence or absence of hydrocephalus, BI, retroflection of the odontoid, Klippel–Feil malformation, and Syr are also evaluated. Hydrocephalus is defined by an Evans index (EI) ≥ 0.30, calculated by dividing the maximal width of the frontal horns by the maximum inner skull distance in the axial T1-weighted MRI at the same slice [40]. BI is defined when the tip of the odontoid process is at least 6 mm above the Chamberlain line [41]. In patients with Syr, the cavity extension in the sagittal plane, the syringo-cord ratio (SCR), and the syrinx–canal index (SCI) were defined before and 6–12 months after surgery (Figure 3). The extension of the Syr was quantified as the number of vertebrae between the superior and inferior limits of the cavity. The Syr maximum anteroposterior (AP) diameter at its widest level was measured on the midsagittal MRI together with the maximum AP diameter of the spinal cord at the same level. In addition, the spinal canal diameter was defined as the maximum AP diameter at the same level at which the maximum AP diameter of the Syr was measured. The SCR was calculated by dividing the maximum AP diameter of the Syr by the AP diameter of the spinal cord at the same level (SCR %: AP diameter of the Syr/AP diameter of the spinal cord × 100 (Figure 3)) [42]. The SCI was calculated by dividing the maximum AP distance of the Syr by the AP of the spinal canal at the same level (SCI %: AP diameter of the Syr/AP diameter of the spinal canal × 100 (Figure 3)) [43]. Postsurgical changes in Syr were classified as improved/disappeared, unchanged, or increased. All patients who underwent PFR had a control MRI 6–12 months after surgery, and clinical and radiological follow-up continued for at least 5 years after surgery.

### 2.2. Indications for Surgery

We recommend surgery for children in the following clinical scenarios: (1) CM-1 asymptomatic patients or patients with no relevant symptoms but with significant Syr, SEP, and BAEP abnormalities, and/or significant sleep study respiratory abnormalities not attributable to other causes; (2) symptomatic patients with refractory headache, Syr, and/or abnormalities upon neurological examination. In patients with hydrocephalus, we routinely monitor ICP first and shunt the patient in the first instance if ICP presents significant abnormalities compatible with active or compensated hydrocephalus [44], reserving PFR for children in whom symptoms persist after the shunt.

### 2.3. Anesthesia Protocol

Surgery is always performed with the patient in a prone position, with the neck slightly flexed, and, in children over one year of age, with the head fixed with a three-pin head holder. The anesthesia management protocol includes balanced general anesthesia. Inhaled induction anesthesia is performed if venous access is difficult. Orotracheal intubation is carried out with a reinforced tube to avoid kinking in the prone position. Special care is taken in the positioning of the patient, avoiding cervical extension. After anesthesia induction, two venous accesses of a caliber appropriate to the patient’s age or central venous access are placed. An isotonic balanced solution is administered at 10 mL/kg/h, with or without glucose, while the dura mater is opened; any additional losses are replaced with the same solution. For postoperative analgesia, we use metamizole, morphine, and anti-emetic agents. Most patients are extubated in the operating room (OR) and transferred to the intensive care unit. Dexamethasone is only used initially (first 24–36 h) to control nausea and vomiting. Sulfamethoxazole and trimethoprim are used as prophylactic antibiotics given during anesthesia induction and followed by three additional intravenous doses.

### 2.4. Posterior Fossa Reconstruction/Surgical Technique

All patients in this cohort were operated on by two of the authors (MAP or JS) using a standard procedure the original surgical technique named ‘posterior fossa reconstruction, PFR’ reported in 1994, with minor modifications described here [15,29]. The head is washed twice: once in the ward before the patient goes to the OR and again after induction of anesthesia, after the patient is positioned and fixed on the surgical table. The operation is performed with the patient in a prone position, with the neck slightly flexed and secured in a Mayfield-type three-pin head holder. The surgical field is then painted with povidone–iodine solution and covered with povidone–iodine-soaked gauze strips for at least three minutes.

**Figure 4:**
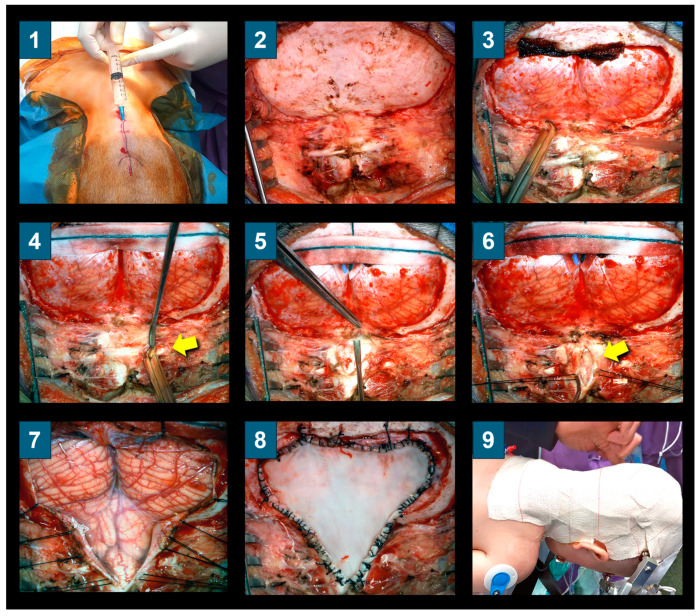
Surgical steps included in the “Posterior Fossa Reconstruction”. (**1**): Patient in a prone position, with the neck slightly flexed and secured in a Mayfield-type three-pin head holder; the surgical field is then painted with povidone–iodine solution and covered with povidone–iodine-soaked gauze strips for at least three minutes; before starting, the surgical incision is infiltrated with local anesthetic with adrenaline to reduce skin bleeding. (**2**): After opening the muscular planes through the nuchal ligament, a subperiosteal dissection is performed to expose the occipital bone, posterior arch of C1, and spinosa and laminae of C2. (**3**): Occipital craniectomy that reaches the lower part of the transverse sinuses at its upper limit. An important point is to properly open the foramen magnum on its two lateral margins. (**4**): Resection of the posterior arch of the atlas at the subperiosteal level (yellow arrow). (**5**): Opening and resecting the posterior atlanto-occipital membrane using a Penfield #4 dissector. (**6**): Beginning the opening of the dura mater, observing that the underlying arachnoid re-expands and appears intact (yellow arrow). (**7**): Completely open dura mater with intact arachnoid and no arachnoid scarring. (**8**): Expansile dural graft placed and closed watertight with continuous non-absorbable suture. (**9**): Compressive dressing, which is maintained for 5–7 days. More images and details of the surgery can be seen in the Supplementary Materials.

The original PFR technique includes three main steps [15]: (1) an extensive suboccipital craniectomy, reaching the inferior parts of both transverse sinuses as the superior limits, with a lateral extension of about 3 to 4 cm on each side of the midline and an extensive lateral bone resection of the FM and C1 laminectomy. Suboccipital craniectomy is carried out with a high-speed drill and bone rongeurs. The FM bone is surgically taken off at either side using bone rongeurs and/or high-speed drills and ball diamond burrs with a head diameter of 3–4 mm until the posterior medial area of both occipital condyles is reached; (2) with the aid of a microscope or surgical loupes, the dura mater is opened in a standard Y-shaped incision and tacked with interrupted sutures aiming to maintain the arachnoid membrane intact; a small opening in the dura is first made in the vertical limb of the dural incision and a Penfield #4 dissector is used to separate the dura from the arachnoid [15]. When the arachnoid membrane is accidentally perforated, low-intensity bipolar coagulation is used to try to seal any holes. If the arachnoid is disrupted and sealing is not feasible, it is opened extensively with sharp scissor dissection to cut arachnoid adhesions. We do not manipulate the tonsils in standard CM-1 without arachnoid scarring; (3) the dura mater is closed watertight. We originally used cadaveric lyophilized grafts, which were substituted in 2001 by a lyophilized allograft (Tutopatch^®^, BIO IMPLANTS Medical S.L, Barcelona, Spain) when cadaveric implants were associated with Creutzfeldt–Jakob disease [45]. The expansile graft is designed to allow a spacious PF to be reconstructed and it is sutured watertight with multiple or continuous 3-0 silk sutures (Figure 4). When the graft is completed, meticulous hemostasis is carried out with copious irrigation to flush out all residual blood and bone dust. A sponge sealant patch (TachoSil^®^, Takeda Austria, Linz, Austria) is used to completely seal the dural graft. We adhere to the guidelines provided by George et al. when using TachoSil [46]. TachoSil is applied to the outer surface of the dura or duraplasty in a single-layer application. The application includes an overlap of at least 8 mm on all sides of the main suture line [46].

**Figure 5:**
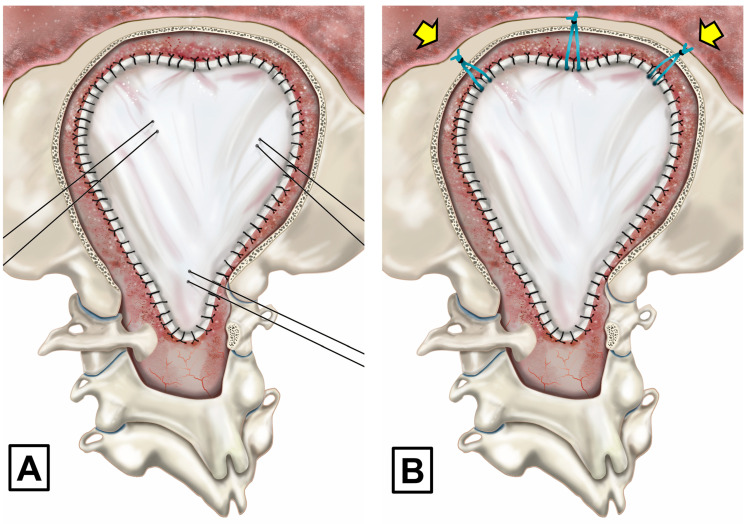
The strategy used to keep the dura mater away from the arachnoid membrane to prevent adherences between the cerebellum and the dural graft. (**A**) The original technique included three to four tenting sutures applied to the dural graft, passed through the muscles, and finally secured to the fascial plane. However, to avoid potential CSF leaks, this maneuver was substituted by tenting sutures from the superior part of the graft secured to small holes made with a drill in the superior nuchal line or, alternatively, to the occipital pericranium (yellow arrows) (**B**).

In the original PFR description, we used three to four tenting sutures applied to the dural graft, passed through the muscles, and secured to the fascial plane (Figure 5). These sutures keep the dura mater away from the arachnoid membrane, preventing adherences from forming and allowing a generous artificial cisterna magna to be formed behind and below the cerebellum. These sutures were the cause of some CSF leaks; therefore, they were discontinued in 2010 and substituted by tenting sutures from the superior part of the graft, secured to small holes made with a drill in the superior nuchal line or to the occipital pericranium (Figure 5).

Two cases in our adult patient cohort presented reversible postoperative ischemic lesions affecting the territory of the posterior inferior cerebellar artery. Vasospasm was identified as the underlying cause of these lesions, stemming from manipulation of the tonsils during subpial resection. Subsequently, we have adopted a protocol involving the application of verapamil-soaked patties positioned along the inferior and medial margins of the tonsils until the conclusion of the dural grafting procedure.

#### Extended Surgery in CM-1 with Arachnoid Scarring and in Patients with CM-1.5

Significant arachnoid scarring is frequently encountered in some patients with CM-1, as well as in those with CM-1.5. In these cases, the arachnoid does not expand upon opening of the dura mater, and the arachnoid is opened, pia–arachnoid adherences are dissected sharply, and the tonsils are either shrunk using bipolar coagulation or partially resected subpially. This is performed to explore the foramen of Magendie and ensure that no membranes or scarring is obstructing it, allowing for adequate CSF flow. Following surgery, the wound is meticulously closed, and a compressive dressing is applied for 5–7 days to minimize the risk of CSF leakage or pseudomeningocele. The Supplementary Materials contains more images and videos demonstrating the surgical method.

### 2.5. Postoperative Adverse Events and Clinical Outcomes

After surgery, patients are always admitted to the pediatric intensive care unit, and, within 36 h following surgery and before discharge to the pediatric ward, a head CT scan is performed to rule out surgical problems. The neurosurgeon in charge of the patient evaluates the clinical result at the time of discharge as well as two months, six months, and one year after the procedure. Afterward, periodic controls every 9–18 months are conducted until adulthood. Every patient has the first follow-up MRI three to six months after PFR. An adverse event (AE) is defined as any event after surgery that results in an undesirable clinical outcome, prolonged patient stay, readmission, or new neurological deficit, requires revision surgery or a new intervention, or contributes to death [47]. Within the 30 days following surgery, any instances of worsening in clinical and/or radiological findings were considered early AEs. Delayed AEs were those that appeared after one month or later post-PFR. As PROSAC is a prospective registry, it was designed to monitor some predefined postsurgical AEs, and, therefore, the report of these does not rely only on institutional administrative databases. After an initial pilot study, the AEs that were routinely monitored and registered in PROSAC within the first 30 days were 1. fever syndrome related to PFR; 2. CSF leaks; 3. hydrocephalus; 4. wound infection; 5. meningitis; and 6. others.

**Surgical outcomes** were defined by both clinical and MRI results. **Clinically**, a child was considered to have had a ‘bad outcome’ if, twelve months after surgery, their clinical symptoms were unchanged or if any permanent neurological worsening occurred after surgery. A good clinical outcome was defined when clinical symptoms were significantly improved and, in patients with Syr, when a significant reduction in length or size was observed in the control MRI. **Neuroradiological failure** or a bad neuroradiological outcome was defined independently of the clinical outcome by the persistence of TH, absence of a neocisterna, unaltered Syr, or a cerebellar slump 12 months after PFR. In children with Syr, when their 12-month MRI revealed no significant improvement in Syr, the radiological outcome was considered bad even if symptoms (headaches, dizziness, etc.) improved.

**Figure 6:**
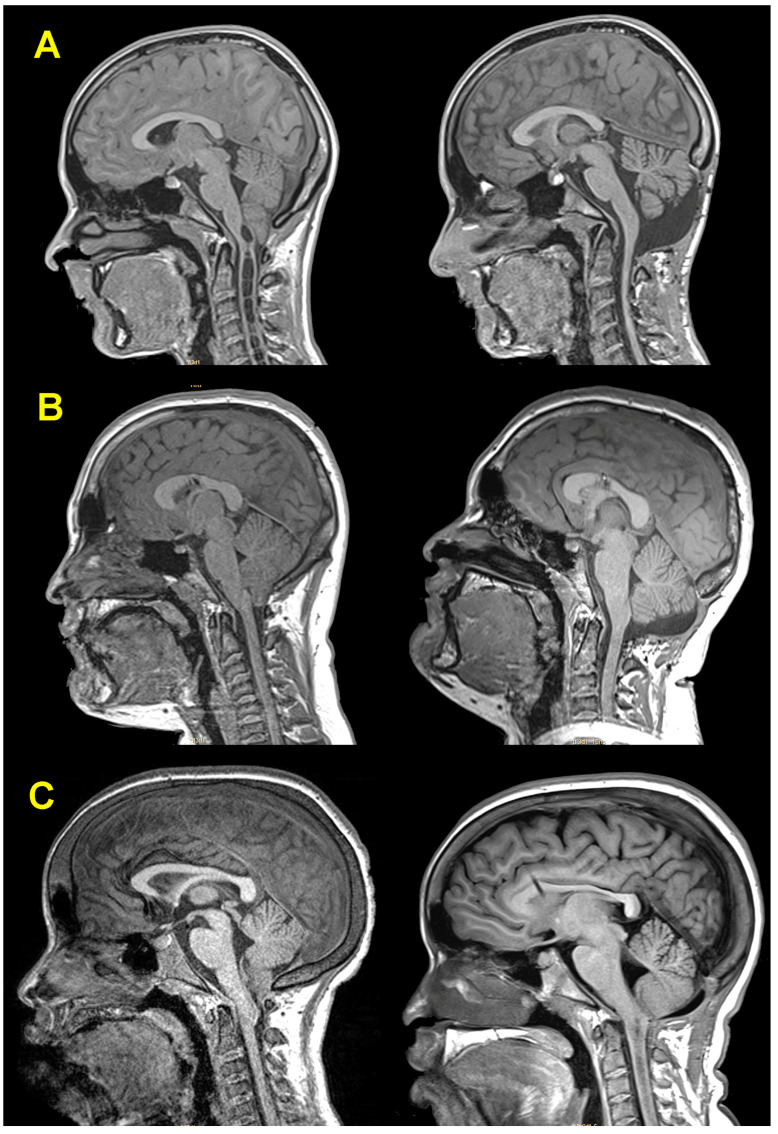
Surgical outcomes. The figure shows three representative patients of those considered as having “very good” (**A**), “good” (**B**), and “bad” (**C**) surgical results. In patient (**A**), there was complete relief of presurgical symptoms and disappearance of the syrinx cavity, with an important improvement of the scoliosis (surgical outcome = very good). In patient (**B**), despite the correct formation of a neocisterna magna after posterior fossa reconstruction (PFR), we considered that the clinical result had not been optimal because the child did not improve from the pre-surgical papilledema. The presence of persistent papilledema, without headaches, despite the ventriculoperitoneal CSF shunt being revised on several occasions, motivated the performance of PFR to be able to implant a lumbo-peritoneal CSF shunt. In this case, the surgical result, which combines the clinical changes with neuroimaging changes, was classified as “good”. Patient **C** is one example of the four patients with a bad surgical outcome: PFR was carried out in a child with sickle cell anemia and headaches precipitated by Valsalva maneuvers; the child had acute hydrocephalus three weeks after the PFR and needed a shunt. The image on the right shows the control MRI showing local arachnoid scarring and the presence of an asymptomatic syringomyelic cavity that was not present before the PFR.

Considering both clinical results and neuroimaging studies, the outcome was qualified as very good, good, or bad. We defined the outcome as (1) very good when symptoms completely disappeared, the children were able to resume their normal life, the one-year MRI showed significant improvement of the Syr—if patients had a Syr before surgery—repositioning of the cerebellar tonsils, the appearance of a neocisterna magna, and no radiological signs of cerebellar slump; (2) good if clinical symptoms disappeared and Syr improved but MRI was suboptimal; and (3) bad when clinical symptoms did not improve or patients developed new symptoms, and also if clinical symptoms improved but, after PFR, the Syr was not significantly reduced, it increased, or the patient presented a new Syr cavity. In brief, when clinical symptoms improved but MRI did not show the expected results, the outcome was always downgraded. Representative patients of each group are shown in Figure 6.

### 2.6. Statistical Analysis

The PROSAC database was designed in PHP code supported by the open-source relational database management system MySQL 5.7 (Oracle Corporation, Austin, TX, USA). The database was hosted in a secured customized intranet running under Debian Linux with anonymized data and access limited to the principal investigators. For the purposes of the study, we queried PROSAC using an SQL command with the previously described inclusion criteria. The records obtained were exported to an Excel file and reimported to R v4.3.3 for analysis [48].

Descriptive statistics were obtained for each variable. Mean and standard deviation were used to describe continuous variables with normal distribution and the median, maximum, and minimum values for continuous variables that were not normally distributed. The Shapiro–Wilk test and inverse probability plot were used to test whether data followed a normal distribution. Percentages and sample sizes were used to summarize categorical variables. To compare between-group differences in categorical variables, χ^2^ statistics or Fisher’s exact test was used as appropriate. Between-group differences were determined by an independent 2-sample *t*-test or the Mann–Whitney U test depending on statistical distribution. Unless otherwise specified, differences were considered statistically significant when *p* < 0.05. Statistical analyses were carried out with R v4.3.3 [48], the integrated development environment R Studio v 2023.12.1.402 [49], and the ‘tidyverse’ package 2.0.0 [50].

#### Data Management

This study was conducted according to the FAIR principles committed to making data and services findable, accessible, interoperable, and reusable. The entire anonymized dataset, metadata, and dictionary are available and can be downloaded from. Sahuquillo, J. (2024). Surgical Outcomes in Chiari 1 and Chiari 1.5 Malformation (pediatric cohort) [Data set]. Zenodo. https://doi.org/10.5281/zenodo.12166149 (accessed on 18 June 2024)

## 3. Clinical Results

### 3.1. Patient Population

Our child cohort (n = 110) consisted of 54 girls (49%) and 56 boys (51%), with a mean age of 9.9 ± 4.7 years (min: 1, max: 18) at the time of surgery. Sixty-six children had CM-1 (60%) while forty-four had CM-1.5 (40%). Eighty-four patients had a primary CM while, in the remaining twenty-six, the CM was associated with other diseases (sickle cell disease, rickets, etc.) (Table 1). Hydrocephalus was diagnosed in 14 patients (12.7%), intracranial hypertension (verified from continuous ICP monitoring) with normal or reduced ventricular size in 8 children (4 with papilledema), and Syr in 48 (43.6%). A retrocurved odontoid was diagnosed in 24 patients (21.8%) and an associated Klippel–Feil malformation was found in just 1 patient. The presence of Syr did not differ between children with CM-1 (42.4%) and those with CM-1.5 (45.5%), χ^2^ = 0.098, *p* = 0.754 (Table 1).

### 3.2. Clinical Symptoms

Of the 110 children who underwent PFR, the CM was an incidental finding in 18 (16.4%). Surgery was offered to 14 children of this asymptomatic group due to the presence of significant Syr. In three patients with shunted CM-associated hydrocephalus, the indication for PFR was poor tolerance to a malfunctioning shunt. In one additional child with CM-1.5, the severity of the brainstem compression and associated cognitive impairment of undetermined origin was the main reason for surgery.

In clinically symptomatic patients (n = 92), children presented symptomatology a median of 24 months before being referred to neurosurgery (min: 1 max: 156), which was significantly shorter in patients with CM-1.5 (Table 1). The most common symptom was headache (n = 71, 77.2%), predominantly occipitonuchal (n = 37, 52.1%), followed by bifrontal (n = 16, 22.58%) or holo-cranial (n = 13, 18.3%). The headache was triggered by Valsalva’s maneuvers in 39 children (55%). Other symptoms were dizziness (n = 24, 26.1%), neck pain (n = 14, 15.2%), fatigue (n = 14, 15.2%), and paresthesia in the upper limbs (n = 14, 15.2%) (Table 1). The type of CM did not influence the percentage of patients with symptoms (χ^2^ = 0.011, *p* = 0.916).

Despite the difficulties involved in performing a thorough neurological examination in some children, this exam was considered normal in most cases (n = 82, 74.5%). The most frequent signs in the remaining 28 patients were sensory disturbances (n = 25, 89.3%), hyporeflexia (n = 6, 21.4%), hyper-reflexia (n = 3, 10.7%), motor weakness in the upper and/or lower limbs (n = 3, 10.7%), nystagmus (n = 3, 10.7%), amyotrophia (n = 2, 7.1%), and Babinski reflex (n = 2, 7.1%). Kyphoscoliosis was observed in eight patients (28.6%). The major clinical symptoms and signs are summarized in Table 1. The presence of Syr or CM type did not influence the number of patients with an abnormal neurological examination.

### 3.3. Results of Neurophysiological Studies

A sleep study was conducted before surgery in 97 of the 110 patients (88.2%), 74 of whom undergoing conventional nocturnal polysomnography and the remaining 23 undergoing cardiorespiratory polygraphy. The criteria of the International Classification of Sleep Disorders 3rd Edition (ICSD-3) considers an apnea/hypopnea index (AHI) ≥ 1 pathological in children [51]. The AHI is the average of the apneic and hypopneic episodes per hour of sleep. In children, an AHI score of 1 to 4.9 events/h is considered mild, 5 to 9.9 events/h moderate, and more than 10 events/h severe. According to this criterion, 61.9% of the patients studied presented abnormal results (n = 60; mild in 45, moderate in 7, and severe in 8). The median AHI was 1.3 (min: 0, max: 90). In 48 of the 60 abnormal sleep studies (80%), central apneas (>50%) predominated over obstructive ones. Furthermore, the study found that the occurrence of apneas did not differ between children with CM-1.5 compared to those with CM-1 (χ^2^ = 1.77, *p* = 0.183).

BAEP was carried out in 94 and SEP in 93 of the 110 patients. The study was not performed on the remaining children due to a lack of cooperation. BAEP presented some abnormality in 18 of the 94 children studied (19%). This percentage was not significantly different between CM types (χ^2^ = 1.06, *p* = 0.304). Twenty-five of the ninety-three patients studied (26.9%) had a SEP alteration in either their upper (median nerve) and/or lower extremities (tibial nerve). The presence of Syr or the CM subtype was not significantly different in children with altered SEPs (χ^2^ = 3.04, *p* = 0.081 and χ^2^ = 0.0007, *p* = 0.980, respectively). Interestingly, 19.6% of children with CM without Syr also presented abnormal SEPs.

### 3.4. Surgical Findings

In PROSAC, we prospectively predefined a way of categorizing the findings observed by surgeons during PFR. The surgeon categorized arachnoid scarring on an ordinal scale: 0 = no arachnoid scarring, 1 = mild arachnoid scarring, 2 = moderate arachnoid scarring without adhesions, 3 = important arachnoid scarring with some adhesions but easy to dissect, and 4 = adhesive arachnoiditis. We also recorded whether the tonsils needed to be coagulated or resected subpially. Moderate and severe arachnoidal scarring was significantly higher in patients with CM-1.5 (χ^2^ = 4.37, *p* = 0.037). In Table 2, we summarize the main surgical findings in both CM categories.

### 3.5. Neuroimaging Findings before and after Surgery

The mean ventricular size before PFR, calculated by the EI, was 0.26 ± 0.03 (min: 0.20, max: 0.34). In eight children who had an EI > 0.30 at the time of PFR, four had a functioning CSF shunt implanted before PFR. The ectopia of the cerebellar tonsils below the FM was highly variable, with a median of 12.5 mm (min: 3, max: 32 mm). As expected, ectopia of the tonsils was significantly greater in children with CM-1.5 than in those with CM-1 (U = 469, *p* < 0.001). Syr was found in 48 patients (43.6%), with the most frequent location being the cervicothoracic region (n = 27). Before surgery, the median SCR was 70% (min: 21.5, max: 90.4%) and the median SCI was 75.8 (min: 33.3, max: 100%).

**Figure 7:**
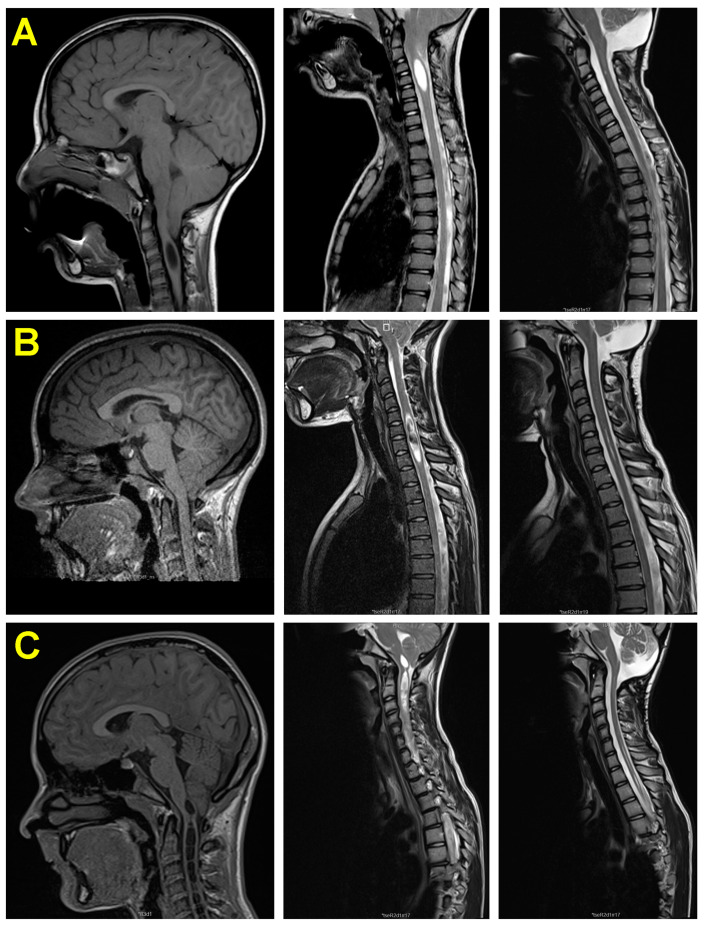
Examples of postoperative syrinx evolution in three patients (**A**–**C**). Each patient’s left and middle images represent presurgical findings, and the right represents postoperative changes in the syrinx cavity. Note that the cavity disappeared completely in patient (**A**) and was reduced to a minimum patency of the ependymal channel in patients (**B**,**C**). There is a new wide pseudo cisterna magna in all three cases.

**Postoperative MRI**. All patients had a complete cranial and spinal MRI to evaluate 6- and 12-month outcomes. In 14 of the 48 (29.2%) patients with Syr, it was undetectable one year after PFR. The dimensions of the Syr significantly improved in 32 of the remaining 34 patients (reduction in length and diameter in 16 patients, diameter in 15, and length in 1). After surgery, the median SCR and SCI in these 34 patients were 36.8% and 56.8%, respectively. These values were significantly lower than before surgery (*p* < 0.001). Figure 7 shows three examples of postoperative Syr evolution.

In one child, Syr remained unchanged, and, in another, it increased. Another child with sickle cell disease, who did not have Syr before PFR, developed Syr a few months after surgery. During the entire follow-up period, which lasted from 12 months up to 17 years, none of the operated children presented signs of CVJ instability.

### 3.6. Postoperative Adverse Events (AEs)

Following surgery, there was no neurological worsening or death among the children. Most children (n = 77, 70%) had an uneventful recovery and were discharged home on average a week after surgery. However, in 33 children (30%), we recorded at least one postoperative AE (Table 2). **Fever.** In all CMs, we closely monitored axillar temperature with a digital thermometer until discharge. Fever was considered when the temperature exceeded 38.0 °C. Fever related to surgery (‘aseptic meningitis syndrome’, AMS) was the most frequent AE in our cohort (n = 25, 22.7%). Fever was diagnosed within the first two weeks after PFR in 17 of the 25 children (68%) and more than two weeks after PFR, when patients were already discharged home, in 8 children. Interestingly, only 2 of the 17 children who had a CSF shunt implanted before PFR presented fever. The fever in these patients had a very predictable pattern: it began 5–15 days after PFR, predominated in the evening, and was clinically well tolerated. It responded very quickly to dexamethasone and required 2–3 weeks of treatment. In some cases, rebound fever occurred after corticosteroids were stopped. In 13 of the 25 children, lumbar punctures were performed, verifying CSF pleocytosis (usually with a predominance of lymphocytes), increased protein, decreased glucose, and no bacterial growth (negative cultures). The condition was very mild in the remaining 12 children, and the diagnosis was made by exclusion. All cases were resolved with dexamethasone therapy. However, the diagnosis of this benign AMS prolongs the hospital stay in some children and requires treatment with low-dose corticosteroids for a varying amount of time, which could range from a few days to several weeks after surgery.

**CSF leaks.** Five children experienced temporary CSF leaks through the surgical wound (4.5%). The wounds were resutured and the leaks resolved. However, in one child, it was followed by bacterial meningitis (coagulase-negative Staphylococci) and required targeted antibiotic treatment. **Surgical wound complications**. One child presented a superficial wound infection, while another had a partial wound suture dehiscence upon removal of the stitches. **Pseudomeningocele.** A total of 11 of the 110 patients (10%) experienced a significant bulging and accumulation of CSF in the posterior fossa, without previous hydrocephalus. These patients required one or more lumbar punctures to evacuate the CSF and local compressions. Temporary administration of acetazolamide was effective in eight patients. The remaining three, however, required the implantation of a ventriculoperitoneal shunt. Three additional children required a delayed ventriculoperitoneal shunt during the first year of follow-up, a median of 56.5 days after the PFR (min: 38, max: 250).

**Other adverse events.** One child presented a significant epidural hematoma of the posterior fossa in the early CT scan, which required surgery. There were systemic complications in three cases: one child presented postoperative croup, which required treatment with corticosteroids, and two had urinary infections. It is noteworthy that even though the surgery was more aggressive in children with CM-1.5, there were no significant differences in the percentage of postsurgical AEs between both groups (χ^2^ = 0.115, *p* = 0.734).

### 3.7. Effectiveness of Surgery and Outcomes

Table 2 provides a summary of the surgical findings and results after PFR. None of our included patients were lost to the one-year follow-up. The one-year clinical outcome after PFR was very good in 103 of the 110 treated children (93.6%), good in 4 (3.6%), and bad in 3 (2.7%). The neuroradiological outcome was categorized as very good in 101 patients (91.8%), good in 5 (4.5%), and bad in the remaining 4 children (3.6%). The final surgical outcome was evaluated one year after PFR via the criteria defined in the methods section. The final surgical outcome was excellent in 101 children (91.9%), good in 5 (4.5%), and bad in 4 (3.6%). In the four children with bad one-year outcomes, the reasons were no changes in Syr in three children and the development of a new Syr in one. Given the relevance of these cases, we will discuss one of them herein and include the individual analysis of two additional children in the Supplementary Materials.

### 3.8. Brief Description and Analysis of the Surgical Failures

**Figure 8:**
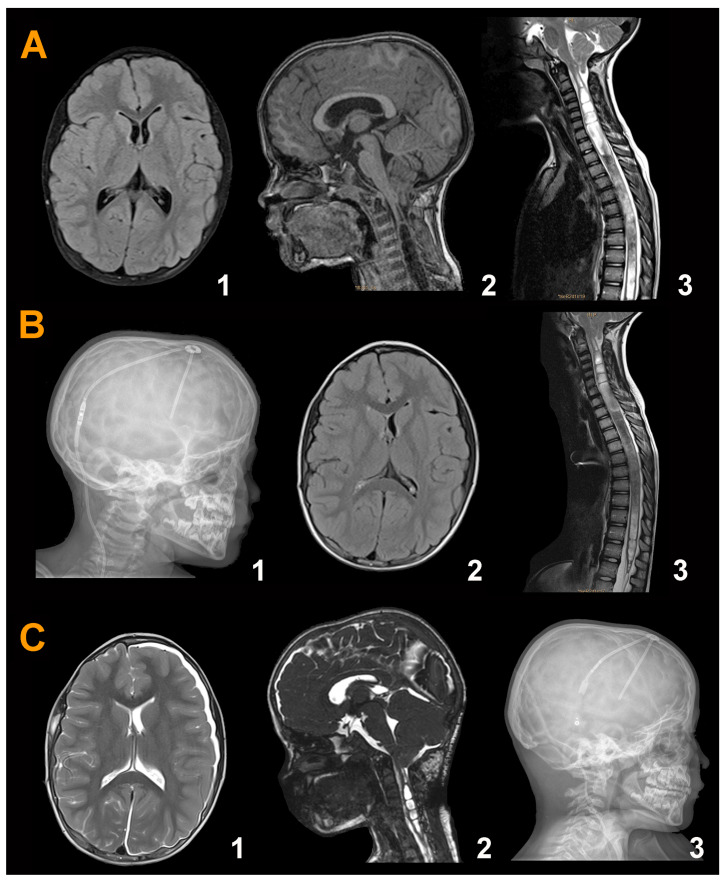
The section analyzing surgical failures contains a summary of the findings in Patient 1. (**A**): Magnetic resonance (MR) findings at diagnosis: (**1**). Normal ventricular size, (**2**) Chiari malformation type 1.5, and (**3**) holomedular syringomyelia. (**B**): Initial therapeutic maneuver with the implantation of a ventriculoperitoneal shunt (**1**,**2**), without changes in the posterior fossa and the syringomyelic cavity. (**C**): Postoperative results after posterior fossa reconstruction, with small supra- and infratentorial hygromas (**1**,**2**) and change of the valve model (**3**). Please refer to the text for more specific details.

**Patient 1.** This six-year-old girl was diagnosed with X-linked hypophosphatemic rickets. She had delayed growth and congenital *geno varus* but no other signs of active rickets. Bilateral papilledema was detected as an incidental finding in the routine study of growth hormone administration. The girl had no history of headaches or other symptoms, and the neurological examination was normal. The presence of papilledema motivated the performance of an MRI (Figure 8A(1–3)), which showed a normal-sized ventricular system (EI = 0.26) with an ectopia of the cerebellar tonsils of 15 mm, associated with a holomedullary Syr. SEPs were abnormal bilaterally and BAEP was normal. In March 2015, the patient received a ventriculoperitoneal CSF shunt (Miethke Paedi GAV^®^ 9/29, Aesculap, AG, Tuttlingen, Germany), which resolved the papilledema without any changes in the MRI (Figure 8B(1–3)). PFR was performed in September 2015 with a complete extra-arachnoidal surgery. In the postoperative period, asymptomatic supra- and infratentorial hygromas were observed (Figure 8C(1,2)). We removed the PaediGAV valve and implanted a Hakim programmable valve (Medos S.A., Le Locle, Switzerland) with an opening pressure of 150 mmH_2_O (Figure 8C(3)) and a low-pressure gravitational compensating device (NMT Neurosciences Implants S.A., Sophia Antipolis Cedex, France). The three-month follow-up MRI showed a very small neocisterna magna and a minor reduction in the Syr, especially in its dorsal segment. However, subsequent follow-up MRI showed an increase in the size of the cervical Syr, so the valve opening pressure was reduced to 100 mmH_2_O. During the following years, the girl was asymptomatic. We offered the family a new surgery; however, they decided to adopt a wait-and-see attitude.

**Figure 9:**
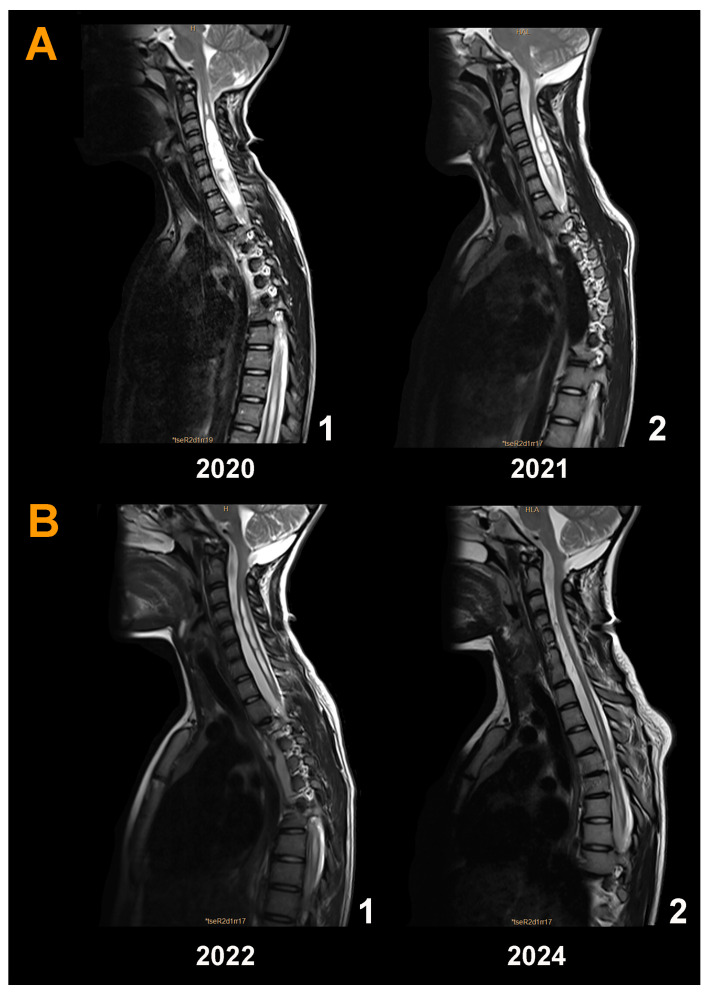
(**A-1**): Increase in the scoliotic curve of the patient presented in Figure 8 (2020). (**A-2**): Results of the second surgery on the posterior fossa with the presence of an artificial cisterna magna and a clear reduction in the size of the syringomyelia (2021). (**B**): Follow-up magnetic resonance studies showing the maintenance of the previous surgical results (2022 and 2024). Please refer to the text for more specific details.

In subsequent years, the girl presented a gait disorder and scoliosis; therefore, in March 2020, we performed a second surgical approach on the PF (Figure 9A(1)). Calcification of the dural graft was found with severe arachnoiditis. We removed the dural graft, and subpial resection of both tonsils was conducted until the foramen of Magendie was visible. Both posterior inferior cerebellar arteries exhibited vasospasm but regained their normal caliber with surgical patties soaked in verapamil. The postoperative MRI showed a significant reduction in Syr and the valve opening pressure was maintained at 200 mmH_2_O without papilledema recurrence (Figure 9A(2)). The presence of the neocisterna magna was maintained over time, as was the reduction in the diameter of the syringomyelic cavity (Figure 9B(1,2)). Cause of surgical failure: we believe that we should have initially implanted a programmable valve instead of a fixed-pressure one to be able to increase the opening pressure and favor the formation of a new cisterna magna. In addition, we believe that we should have been more aggressive in the first surgery, where the arachnoid should have been opened together with a reduction in the cerebellar tonsils to ensure the correct opening of the foramen of Magendie. Additional children’s results are described in the Supplementary Materials.

## 4. Discussion

Herein, we present the one-year outcomes of a large single-center series of prospectively compiled pediatric patients with a CM1 or CM-1.5 uniformly treated with PFR. In this study, we defined the surgical technique, assessed the clinical and neuroradiological outcomes at one year post-surgery in a cohort of 110 children, and developed a composite clinical and neuroradiological score to assess the final surgical outcome. Additionally, we conducted an in-depth analysis of the factors contributing to surgical failure and presented the logic behind the adoption of PFR as an expanded approach for managing children with CM-1.5. PFR goes beyond merely decompressing the FM: it encompasses reconstructing the PF with the dual objective of relieving pressure on the neural elements and restoring both the volumetric capacity of the PF and CSF circulation around the FM. This surgical strategy aims not only to address the immediate symptoms but also to rectify the underlying structural and functional disturbances, offering a more comprehensive solution to patients with CMs.

### 4.1. Should Asymptomatic Children with Syr Have Surgery?

In the present study, 43.6% of patients had Syr, without any significant difference between children with CM-1 and those with CM-1.5. The prevailing hypothesis for the cause of CM-related Syr was proposed by Oldfield et al., 1994. According to this theory, herniation of the cerebellar tonsils restricts or completely obstructs the flow of CSF around the FM, leading to an increase in cervical subarachnoid pulse pressure. This sequence of events culminates in the development of Syr [52,53,54,55]. In individuals without CSF obstruction at the FM, each systolic pulse triggers a rapid movement of CSF from the basal cisterns to the upper portion of the spinal canal. This movement accommodates the increase in volume that occurs with each systolic wave [52]. In CMs, the cerebellar tonsils plug the subarachnoid space, blocking CSF movements. In this situation, the tonsils move downward with each systolic pulse, acting as a piston on the partially isolated spinal canal and inducing a systolic pressure wave peak that acts on the surface of the spinal cord, increasing the bulk flow of CSF—flow from a high-pressure to a low-pressure area —toward the central canal through the Virchow–Robin spaces [52].

This theory is the most widely accepted and replaced the initial theories of Gardner or Williams, who proposed the hydrodynamic and cranial–spinal pressure dissociation hypotheses, respectively, to explain Syr based on CSF flow via a patent communication between the fourth ventricle and the Syr [56,57]. Heiss et al. proposed that the impaired CSF displacement across the FM during the cardiac cycle and the increase in amplitude of the CSF pressure wave drives CSF into the spinal cord from its surface through the perivascular spaces [53,54,55,58]. At our institution, we propose surgery for all children with significant Syr-associated CM-1 and CM-1.5, even when they are asymptomatic. This recommendation contradicts the consensus reached at the ICC-CM, in which 82.4% of physicians agreed that surgery is not always necessary in children with asymptomatic Syr [13]. In this consensus conference, it was stated that “*In asymptomatic children with incidentally discovered CM-1 and syringomyelia, surgery is indicated in cases of syrinx larger than 5–8 mm, and smaller syrinx increasing in size*” [13]. When children with CM-related Syr have a normal neurological examination and no symptoms, neurosurgeons are reluctant to perform surgery because of the potential risks involved, as well as the divergence between neuroimaging results and clinical evaluations. This cautious approach is reinforced when neurophysiological tests show normal findings, even in large Syr.

The natural evolution of untreated CM-related Syr in children is uncertain and we are not aware of any cohort of children large enough and with a long-term follow-up to extract any conclusion. In 17 children with CM-related Syr managed without surgery and a median follow-up of 2.3 years, Singhal et al. reported that Syr was unchanged or diminished in size in 15 patients (88%). Nevertheless, Syr increased in size in two children, one of whom had clinical worsening [59]. Despite the absence of strong evidence, we hold the view that the detection of any significant Syr in children warrants a discussion with families about the risk/benefit ratio of surgical intervention to address the underlying cause. Our belief is based on our observation of the often severe long-term effects of untreated Syr in adults or children who are transitioning into adulthood. There is increasing evidence that, in CM-associated Syr, the appearance of symptoms and neurological signs depends on the time of follow-up [60,61,62], with the additional risk that, when neurological deficits appear, they cannot be reversed by surgery [63,64]. Other authors have stated that patients should undergo operations early on, before spinal cord lesions become irreversible [62,64,65]. Aghakhani et al., in a long-term follow-up of 157 adult patients with CM-related Syr, found that factors associated with a higher probability of improvement or stabilization included young age at surgery [63]. Older age, arachnoid scarring, and signs of long tract dysfunction—corticospinal or sensorimotor signs—were associated with poor clinical outcomes [63]. In most cases, CM-related Syr shows progression over long-term follow-up. We believe that the risk/benefit ratio of treating children with Syr is favorable because it addresses the primary etiological factor and diminishes the anxiety experienced by families undergoing repeated MRI and neurological examinations for follow-up. In our cohort of 48 surgically treated children with Syr, in 46 cases, Syr completely disappeared (n = 14) or was reduced to a minimal ectasia of the central canal (n = 32). Two other cases in which Syr did not improve are discussed in the surgical failure section and the Supplementary Materials. Our results show that, in line with other research, successful surgery that restores CSF dynamics at the FM level is the best treatment approach for CM-related Syr [52,53,58,66,67].

### 4.2. Decompressing versus Reconstructing the Posterior Fossa

It is currently accepted that an underdeveloped and consequently volumetrically diminished PF is the primary etiology of CMs [8,19,20,21,22,24,25,26,27]. Urbizu et al., in a case–control study, showed that a mathematical model based on PF morphometric analysis could predict a CM-1 diagnosis with 93% sensitivity and 92% specificity, regardless of the degree of tonsillar herniation [68]. We believe that the surgical technique used in the treatment of CM is important for achieving the best neuroradiological results and clinical long-term outcomes. In addition to decompressing the PF, surgery should aim to expand it volumetrically. However, most studies analyzing the surgical outcomes of CMs tend to neglect the neuroradiological outcomes obtained with the various techniques used to decompress/restore the volume of the PF.

A limited number of publications have explored the neuroradiological outcomes observed after PF surgery. Duddy and Williams, in their seminal work, quantified cerebellar slump after PF surgery, and their paper is often referenced to justify performing a minimal craniectomy as a means to prevent cerebellar ptosis [16]. In their report, the authors suggested that “*The size of the craniectomy was deliberately limited to about 4–5 cm in height and about 3–4.5 cm in width in order to limit the tendency of the cerebellum to slump*” [16]. Duddy and Williams assessed the MRI results of 17 patients with treated CM-1, and they reported that the “*Elevation of hindbrain structures was uncommon, and when seen was of small magnitude (2 mm or less) and may be within the margins of error*” and also that “*Descent of the hindbrain occurred more frequently than had been expected*” [16]. There is no doubt that slump is the primary cause of unsatisfactory long-term clinical outcomes and that the sinking cerebellum maintains the craniospinal pressure dissociation at the CVJ, causing Syr to progress (Figure S7 in Supplementary Materials). However, as we discuss here, the size of the suboccipital craniectomy is not the crucial factor favoring cerebellar ptosis. Duddy and Williams, in the same paper reporting cerebellar ptosis, remarked that “*Slump was also greater in patients with a small artificial cisterna magna and little CSF space below the cerebellum, and was less amongst those with a large bulging cisterna magna* (*p* < 0.02)” [16]. To the best of our knowledge, and paradoxically, these authors were the first to describe how a sizable artificial cisterna magna, and the buoyant force that it generates, could prevent the cerebellum from sinking into the skull after a suboccipital craniectomy.

### 4.3. The Buoyant Brain: A Neglected but Fundamental Property of Neural Tissue

The concept of the ‘buoyant brain’ presents an intriguing perspective on the often overlooked intrinsic property of neural tissue: its buoyancy. The capacity of an object to float in a fluid is known as buoyancy, and it is strongly related to its specific gravity (SG). SG is a unitless measure of the ratio between the density of a substance in comparison to the density of pure water at a known temperature and atmospheric pressure. A substance will float if its SG is lower than that of the liquid that it is submerged in [69]. The buoyancy of the brain can be attributed to its composition and the specialized fluid environments in which it operates: the CSF. The Archimedes principle, a cornerstone of fluid mechanics, states that any fluid exerts a buoyant force on an object wholly or partially submerged in it, and the magnitude of the buoyant force equals the weight of the fluid that it displaces [69]. Levin et al. calculated that the SG of normal human CSF at 37 °C ranges between 1.0063 and 1.0075 [70]. The brain’s SG has been less studied but radiological attenuation is linearly correlated with SG in human tissue, and this property allows for measuring the volume and SG of the brain in vivo [71]. Lescot et al., using a CT scan, showed that the SG of the CSF in healthy controls was ~1.0219, the SG of the brain was 1.0335, and the cerebellum increased to ~1.0375 [71]. Because of the similar SG of the brain and CSF, the human brain has what is called ‘neutral buoyancy’ because of its suspension in the CSF, and this property prevents the brain from compressing the neural tissue and its blood vessels against the internal surface of the skull.

In humans, the adaptation to bipedalism causes gravitational changes in the position of the brain and cerebellum while moving from lying down to sitting or standing. However, these changes are insignificant as long as the volume of CSF remains constant. Lee et al. observed that, in healthy children, the position of the cerebellar tonsils did not change significantly after changing from supine to standing using a 0.25 T MRI [72]. This makes sense as the brain’s neutral buoyancy keeps gravitational changes negligible. A different study that used a CT scan with 0.5-mm slices—much smaller than MRI slice thicknesses—found that, when sitting, the cerebellar tonsils in healthy persons shifted 2.10 ± 0.86 mm to the spinal canal [73]. Even though this movement is not relevant, it is conceivable that, without a cisterna magna, as occurs in CMs, there is a significant reduction in cerebellar buoyancy. This reduced/absent buoyancy might lead to a greater displacement in the cerebellar tonsils induced by gravitational forces when moving to an upright position. Three lines of evidence indicate that this may be the case: 1. in situations where CSF volume has been significantly reduced, as occurs in patients with spontaneous intracranial hypotension, the loss of CSF buoyancy can induce neurological worsening as a result of the posture-dependent descent of the brainstem provoked by opening the hypovolemic intrathecal space to atmospheric pressure [74]; 2. craniospinal pressure gradients can induce TH without any increase in ICP in the presence of iatrogenic spinal CSF leaks that have been described after insertion of lumbar drainages, lumbar disc surgery, lumboperitoneal shunting, or even intrathecal pump placement [75,76,77]. Significant spinal CSF leaks generate a significant pressure gradient across the FM, which provokes acute TH, neurological deterioration, or even death if the CSF leak is acute and severe, as described in some cases induced by lumbar drainage [78,79]; 3. cerebellar sinking into the craniectomy is the most common cause of persistent or even progressing Syr in CM patients in whom PF surgery has been performed using any technique and in which a CSF cushion is not observed below the cerebellum, thus creating the conditions for buoyancy, as suggested by Duddy and Williams [16].

### 4.4. What Constitutes a Successful Surgical Outcome in CM?

Defining a favorable surgical outcome involves assessing patient-reported outcomes and objective clinical improvements post-surgery. The main challenge in assessing surgical outcomes across series of patients with CMs—both adults and children—is that most studies focus primarily on reducing preoperative clinical symptoms and/or Syr; however, they do not reach a consensus on what constitutes a ‘good neuroradiological’ outcome or even if neuroradiological results, apart from Syr, should be considered in the assessment of the outcome. There is sufficient evidence to conclude that if preoperative symptoms are resolved but the PF does not have a favorable neuroradiological result, then both symptoms and Syr could recur. We believe that this is of particular importance when discussing the outcomes of different surgical procedures used to treat CMs.

A certain number of patients, particularly children, with CM-related Syr may improve with only bone removal and no duraplasty. However, retrospective studies consistently show that superior results in terms of clinical outcomes and morphological changes in syrinx dimensions are achieved when the dura is opened and duraplasty is performed [80]. In the pediatric population, some authors have suggested a ‘minimally invasive management’ with only occipital bone removal, C1 laminectomy without dura mater opening, or with only delamination of its outer layer [18,81]. Caldarelli et al. used this approach in 30 children and they reported that, with this less aggressive approach, most patients improved clinically—with a mean follow-up of 4.6 years—despite minor or no modifications in the position of the cerebellar tonsils on the postoperative MRI [18]. Caldarelli et al. remarked that “*In contrast with the good clinical outcome, the neurological improvement was less rewarding, as the anatomical features of CM-I remained almost unchanged in most cases*” [18]. Munshi et al. conducted a retrospective study involving 37 patients, 11 of whom were children. The study compared the outcomes of PF with and without duraplasty and calculated the area of CSF behind the cerebellum. The findings indicated that patients who had a significant decrease in Syr following surgery all showed an increase in the area behind the cerebellum, though, in certain cases, patients without a duraplasty could still achieve this goal (Munshi, Figure 1) [80]. They noted that the majority of patients with a significant decrease in Syr “exhibited an increase in CSF space behind the cerebellum”, which is consistent with our findings, and remarked that the “*…correlation between an increase in the size of postcerebellar CSF space and a decrease in hydromyelia with an improvement in symptoms may be a key observation in this group*” [80].

We stress that even while a child’s clinical outcome appears to be satisfactory, it can be difficult to claim that the condition resolved if there are no apparent modifications in the PF, particularly if there is no discernible CSF below the cerebellum on postoperative MRI. In these children, the outcome should be reassessed at least five years after surgery. Our findings in both adults and children indicate that most patients relapse after a few years and that Syr typically progresses when postoperative MRI fails to demonstrate any discernible remodeling of the PF or when there is cerebellar slump (unpublished results). In our series of 334 children, 17 cases were treated at another institution. These patients were excluded from the present series; nonetheless, we agree with Talamonti et al. that, when a treated patient with CM requires reoperation, the more frequent reasons are: 1. an incorrect initial indication or surgical technique; 2. postoperative complications; and/or, 3. ineffective surgery that can be improved by a more invasive procedure [82].

The best biomarkers of a successful CM surgery include a decrease in tonsil descent, a significant decrease in Syr, and the appearance of a neocisterna magna underneath the cerebellum. The restoration of an artificial cisterna magna indicates that the CSF is free to flow around the FM, and provides the necessary conditions for creating the buoyant force that keeps the cerebellum from slumping. By acting as a depulsating chamber, the artificially constructed cisterna magna reduces the amount of intracranial pulse pressure transmitted to the spinal canal. This was the initial purpose of our suggested PF ‘reconstruction’ [15,29]. Our surgical findings reported that using less aggressive procedures was insufficient to prevent cerebellar sinking and achieve a favorable long-term clinical outcome. This clinical–neuroradiological approach has been used in this study to assess the final surgical outcome one year after PFR by using the criteria defined in the methods section.

The final surgical outcome was excellent in 101 children (91.9%), downgraded to good in 5 children (4.5%) because of a suboptimal MRI despite improvement of clinical symptoms, and bad in 4 cases (3.6%). The poor outcome in these latter four children was primarily due to the insufficient volumetric expansion of the PF—sufficient CSF behind and below the cerebellum—even after a craniectomy of similar size. The lack of a CSF cushion underneath the cerebellum failed to re-establish cerebellar neutral buoyancy, leading to cerebellar ptosis that either did not change or worsened the Syr. The results section and Supplementary Materials provide a description and illustrations of some of the children who were included in the bad outcome category. We believe that future clinical trials should consistently include both clinical and MRI outcomes to assess the effectiveness of any surgical technique.

### 4.5. Does Cerebellar Slumping Become More Likely after Large Craniectomies?

Symptomatic cerebellar ptosis following PFD in CMs was first described by Williams in 1978 [83]. At the end of his landmark study, he said that “*Decompression of the posterior fossa may have to be limited by the consideration that the cerebellum may continue to slump and become impacted within the bone defect as it previously was in the foramen magnum*” [83]. In 1992, Duddy and Williams reported the occurrence of cerebellar ptosis following a large craniectomy for CM-1, in which the dura mater was left open [16]. The risk of a sinking cerebellum was confirmed by Bartzdorf, another pioneer in the treatment of CMs, who considered that the higher incidence of cerebellar ptosis in Williams’ series was probably a consequence of leaving the dura mater open and not using a dural graft [84]. In another study, the same group reported the analysis of 47 patients who underwent reoperation after Chiari decompression, concluding that “*The most common indication for revision Chiari decompression was a large craniectomy resulting in cerebellar ptosis*” [85]. Interestingly, one of the examples that Zarrin et al. reoperated on (Figure 2) in their publication had a full resolution of significant CM with a large cisterna magna, which is comparable in size to the dimensions that we report here [85].

In light of these findings, as well as the experience of early CM neurosurgeons, it is challenging to say with confidence that an extensive craniectomy is safe as long as the cerebellum’s buoyancy is effectively restored. In our 1994 pilot study, we demonstrated that a large suboccipital craniectomy did not lead to the development of cerebellar ptosis [15]. The ten patients included in our pilot study were followed for a mean of 10 ± 2.0 years and both the clinical and neuroradiological outcomes were stable when discharged from follow-up (unpublished results). From our perspective, the absence of an artificial cisterna magna beneath the cerebellum—which offers sufficient buoyancy to prevent cerebellar sagging—is the cause of the sinking cerebellum rather than the extent of the suboccipital craniectomy. Children may have the ability to expand the dura mater after a bone decompression alone but adults rarely do [80]. However, in our opinion, it is hard to anticipate which children will be able to rebuild a functioning cisterna magna when surgery is limited to bone removal. Furthermore, we believe that children with CM require much more complex reoperations due to arachnoid and dura scarring, even in cases where the dura was not opened during the initial surgery. The increased thickness of the dura is thought to be the result of the inflammatory response triggered by the manipulation of the dura mater (see Supplementary Information). For this reason, if surgery is considered for a child, we prefer to begin by offering the family a PFR.

We believe that this view is supported by the data from this cohort, as well as the study of our surgical failures. In patients with a prior CSF shunt, the inability to generate a sufficiently large cisterna magna is a significant factor to consider in order to avoid poor neuroradiological outcomes. We have changed the way that these patients are treated over the last few years. To lower the shunt’s flow before surgery, we currently always employ a programmable valve in CMs and hydrocephalus in conjunction with a gravitation control device. The valve is reprogrammed after PFR once the control MRI demonstrates a consistent CSF cushion beneath the cerebellum.

### 4.6. Postoperative Adverse Events

Considering that the volumetric enlargement needed to restore cerebellar buoyancy is rarely achieved in children without opening the dura mater and is largely ineffective in adults, the argument regarding whether this approach is still necessary to treat CMs seems to be outdated. While randomized controlled trials are the gold standard for assessing interventions, the inclusion of non-randomized studies can offer additional insights, especially in situations where randomized trials may be challenging or unethical. Several studies, systematic reviews, and meta-analyses have addressed this topic; however, none of the systematic reviews detected any randomized clinical trial [86,87,88]. Nevertheless, these studies help to provide an overview of postoperative complications in children undergoing surgery for CMs. There is inconsistency in the reporting of postoperative complications across studies investigating CM surgery. In the largest retrospective review of papers published in English, Arnatutovic et al. identified 145 operative series of patients with CM, primarily from the United States and Europe [89]. Complications were reported in only 41% of all studies and 56% of the pediatric studies; however, as emphasized by the authors, “*It is unclear whether patients in the remaining 59% of the series did not have complications or simply did not report them*” [89]. Therefore, in summarizing retrospective studies, as expected, the true complication rate is unreliable. In the same extensive study, the mortality rate was 11%, without any significant difference between the pediatric and adult series [89], and the most reported postoperative complications were CSF leak, pseudomeningocele, aseptic meningitis, wound infection, meningitis, and neurological deficit [89].

Twelve relevant studies that treated pediatric patients with CMs, both with and without a dural graft, were included in the systematic review of Lu et al. [88]. They included 1492 pediatric patients treated via PFD with duraplasty and compared them with 1963 pediatric patients treated with a completely extradural approach. When duraplasty was performed, there was a greater overall clinical improvement and a longer hospital stay, but more postoperative complications than with the single extradural procedure [88]. The examination of surgical complications revealed an incidence of 11.8% with the less invasive extradural technique and 15.6% with duraplasty [86]. However, since it was not mentioned in any of the included papers, the neuroradiological results of the two groups were not analyzed; therefore, no conclusion could be reached regarding the number of patients with good surgical outcomes, as defined herein.

In our series, there was no neurological worsening or death among the children, and most (n = 77, 70%) had an uneventful recovery and were discharged home on average a week after surgery. Nonetheless, we recorded at least one postoperative AE—most times a non-serious AE—in 30% of our operated children, which will be examined on an individual basis (Table 2).

#### 4.6.1. Aseptic Meningitis Syndrome (AMS)

The most frequent postoperative AE in our cohort was the diagnosis of fever associated with AMS (n = 25, 22.7%). AMS is a frequent and well-known phenomenon after PF surgery both in adults and children that requires frequent CSF sampling and systemic steroids. Finlayson and Penfield provided a thorough description of this clinical condition in 1941 [90], and it was later named ‘posterior fossa syndrome’ [91]. In general, the term AMS is preferred to avoid confusion with other clinical conditions that may appear after surgery on the PF [92,93]. Clinically, AMS is defined by the presence of a fever that spikes and is usually more noticeable in the evening. It can also occasionally be associated with headache, nausea, vomiting, and some neck stiffness; however, CSF cultures are always negative.

In a cohort of 50 children with PF pathology who underwent surgery, Carmel et al., in 1974, reported that the vast majority (70%) had AMS [93]. Other authors have observed an AMS incidence following infratentorial surgery ranging from 3 to 70% [89,92,93,94]. The frequent inclusion of cerebellar tumors in studies, which routinely receive postoperative corticosteroids, and the diversity of the types of lesions included can bias the true incidence. AMS can occur in the initial days following surgery; however, it becomes more common after a week. In patients who received steroids, AMS appears when withdrawing or reducing the dose. AMS is quite benign and usually a well-tolerated condition but, in some cases, it is accompanied by headaches with nausea and vomiting, subcutaneous accumulation of CSF, and a bulging wound [90,92,93]. When taking steroids at higher doses, the symptoms rapidly disappear, and fever relapse can be prevented by gradually reducing the steroid dosage [92]. If we refer specifically to CM surgery, the incidence of AMS is also highly variable, ranging from 3 to 50% [89,94,95]. Some authors have suggested that the type of material used for the dural graft may contribute to AMS. Autogenous tissues, being nonimmunogenic, nontoxic, easily accessible, and cost-effective, offer certain advantages. However, in practical terms, securing an autologous graft of an appropriate size for our surgical needs, such as pericranium or fascia lata, often necessitates enlarging the cranial incision or making a second incision in the patient. On the other hand, compared with adult tissues, children’s tissues tend to be finer and more fragile. Taking these factors into account, we decided to employ acellular bovine pericardium grafts, which enable us to work with a very large size (Figure S1 in Supplementary Materials) and with macroscopic properties that closely resemble those of the dura mater. Furthermore, no graft type has been proven to be superior to another as they have all been related to postoperative issues, particularly the emergence of AMS.

Due to extensive research in both animal and clinical investigations, an acellular collagen matrix dural graft—a dural substitute generated from bovine, porcine, or equine tissues—is frequently used [96]. Indeed, in all the patients presented here, we used a bovine dural graft. Farber et al. suggested that AMS was significantly greater in patients who underwent duraplasty using xenografts compared with allografts [95]. Another retrospective study (n = 81) by Lee et al. found a strong correlation between the use of a porcine dural graft and a higher risk of pseudomeningocele [96]. Before being accepted, these results should be reproduced in at least one case–control study to support the idea that the materials used for duraplasty have a significant impact on AMS.

The frequency of AMS in our cohort (22.7%) was similar to that reported by Chen et al. In a retrospective study in adults, they observed an incidence of 27.1% in adult CM-1 patients in whom autologous pericranium or ‘artificial’ dura was employed for duraplasty [94]. In our cohort, the duration of this condition, as described by others, lasted from a few days to several weeks, in some cases even exceeding two months [92,93,94]. We closely monitor fever in CM surgery to identify AMS early, and this closer monitoring may partially explain the high AMS rate reported in this paper, which is comparable to those described by others [92,93]. The cause of AMS is still unclear, although it was previously identified years before dural plasties and sealants were employed. These contributing factors included arachnoid membrane opening and the presence of irritants, primarily the byproducts of degenerated blood from the surgical bed that came into contact with the arachnoid.

Finlayson and Penfield reproduced AMS in cats by injecting the blood of the surgical bed aspirated one week after intracranial surgery into the cisterna magna of the same animal from which the degenerating blood was obtained [90]. A precise proteomic investigation of the CSF of patients undergoing surgery, particularly those with CMs, is still lacking; however, the most likely cause of aseptic meningitis is the presence of blood breakdown products in the CSF.

In 1974, Carmel et al. reported a 70% incidence of aseptic meningitis in a consecutive series of children undergoing a PF operation [93]; in a second cohort thirty years later, they observed that this incidence was significantly reduced to 30% with the systematic use of corticosteroids in the postoperative period [92]. In conclusion, many patients who have had infratentorial surgery—including CMs—in which the dura mater was opened develop this common but typically benign condition. Finlayson and Penfield’s 1941 groundbreaking study provides, in our opinion, the most accurate explanation of the clinical impact of AMS on patients: they said that AMS *“...is troublesome rather than dangerous, for it prolongs the convalescent period by two to six weeks, without leaving serious residual complications”* [90].

An interesting finding in our cohort was that only 2 of the 17 children in our series who had a functioning ventriculoperitoneal CSF shunt at the time of PFR presented AMS in the postoperative period. We hypothesize that the shunt favors the clearance of CSF and, therefore, the clearance of ‘irritating’ molecular elements, regardless of what they are.

We believe that a randomized clinical trial should be conducted to assess the efficacy and risk–benefit of routine postoperative corticosteroid use for at least two weeks, with subsequent tapering over approximately one month compared with only treating patients who develop the syndrome. Such a trial would afford a comprehensive assessment of the procedure’s effectiveness in mitigating the incidence and impact of AMS in CM surgery.

#### 4.6.2. CSF Leaks and Pseudomeningocele

Five children experienced temporary CSF leaks through the surgical wound (4.5%), which resolved after resuturing. However, this was followed by bacterial meningitis in one child, who required targeted antibiotic treatment. A total of 11 of the 110 patients (10%) experienced significant bulging and CSF accumulation in the PF, without previous hydrocephalus. These patients required one or more lumbar puncture to evacuate the CSF along with compressive dressings. Three of them required the implantation of a ventriculoperitoneal shunt.

It should be noted that, even though surgery was more aggressive in children with CM-1.5 than with CM-1, there were no significant differences in the incidence of postsurgical AEs between both groups (χ^2^ = 0.115, *p* = 0.734).

## 5. Study Limitations

A limitation of the present study is that the results derive from a single center, with PFR being performed by only two neurosurgeons. This aspect, which is in itself a strength that reduces heterogeneity, has the limitation that the results cannot be generalized unless a similar surgical technique is used. An additional limitation is that we only report the 12-month outcome of our children; a longer follow-up of at least 5 years would be required to consider these results as permanent. These data are already available for a substantial number of long-term follow-ups, and we can confirm that the findings described herein continue to be stable (unpublished results).

## 6. Conclusions

The treatment of CM-1 and CM-1.5 in children with PFR in order to achieve a correct neocisterna magna to significantly enlarge the volume of the PF and recreate the CSF environment that generates buoyancy of the cerebellum is associated with a high percentage of excellent and good clinical results and with a good volumetric enlargement of the PF evaluated at one year post-surgery. Aseptic-meningitis-related fever was the most frequent AE within the first month after surgery; however, in all cases, it resolved with pharmacological treatment without any sequelae. The inability to achieve a good result consistently stemmed from the slump of the cerebellum into the craniectomy. This descent was attributed to the failure to recreate sufficient CSF fluid below the cerebellum to counteract the impact of gravitational forces on the hindbrain.

## Figures and Tables

**Table 1:** Demographic and clinical data in the total cohort of patients and the subtypes of Chiari malformation (CM).

		CM Subtypes		
	Total Cohort (n = 110)	CM-1 (n = 66)	CM-1.5 (n = 44)	*p*
Sex, boys (n/%)	56 (51%)	34 (51.5%)	22 (50%)	0.876
Age, years (mean ± SD)	9.9 ± 4.7	9.8 ± 4.9	10 ± 4.4	0.843
Tonsillar herniation, mm *	12.5 (3–32)	10 (3–30)	17 (7–32)	**<0.001 ^†^**
Isolated CM	84 (76.4%)	46 (69.7%)	38 (86.4%)	
CM and other medical conditions (n)	26 (23.6%)	20 (30.3%)	6 (13.6%)	**0.044 ^†^**
- PIK3CA-related overgrowth	4	2	2	
- Autism spectrum disorder	4	3	1	
- Sickle cell syndromes	4	3	1	
- Hypophosphatemia rickets	3	3		
- RASopathy (MAPK syndrome)	3	2	1	
- Epilepsy	2	2	-	
- Other **	6	5	1	
**Associated problems**				
Syringomyelia	48 (43.6%)	28 (42.4%)	20 (45.5%)	0.754
Hydrocephalus	14 (12.7%)	10 (15%)	4 (9%)	0.350
Intracranial hypertension ***	8 (7.3%)	7 (10.6%)	1 (2.3%)	0.198
Retroflexed odontoid	24 (21.8%)	13 (19.7%)	11 (25%)	0.509
**Clinical symptoms and signs**				
Symptomatic patients	92 (83.6%)	55 (83.3%)	37 (84.1%)	0.916
Time of evolution, months *	24 (1–156)	24 (4–132)	12 (1–156)	**0.010 ^†^**
- Headache	71 (77.2%)	44 (80%)	27 (73%)	0.566
- Dizziness	24 (26.1%)	17 (31%)	7 (19%)	0.320
- Neck pain	14 (15.2%)	9 (16.4%)	5 (13.5%)	0.466
- Fatigue	14 (15.2%)	9 (16.4%)	5 (13.5%)	0.664
- Paresthesia	14 (15.2%)	8 (14.5%)	6 (16.2)	0.500
Abnormal neurological examination	28 (25.5%)	19 (28.8%)	9 (20.5%)	0.299
- Sensory disturbances	25 (89.3%)	16 (84.2%)	9 (100%)	0.642
- Reflex abnormalities	8 (28.6%)	6 (31.6%)	2 (22.2%)	0.368
- Kyphoscoliosis	8 (28.6%)	6 (31.6%)	2 (22.2%)	0.667
- Motor weakness	3 (10.7%)	1 (5.2%)	2 (22.2%)	0.563

* Median (minimum–maximum). ** CM associated with another medical condition: other: CM-1: craniosynostosis, shunt hyperfunction in an arachnoid cyst, Kabuki syndrome, alcoholic fetal syndrome, and Jarcho–Levin syndrome; CM-1.5: hyper-IgE syndrome. *** Intracranial hypertension with normal or reduced ventricular size **^†^** Statistically significant differences between different groups of CM-1 vs. CM-1.5, *p* ≤ 0.05. **CM-1**: CM type 1; **CM-1.5**: CM type 1.5; mm, millimeters.

**Table 2:** Surgical findings and results after posterior fossa reconstruction (PFR) in the total cohort of patients.

		CM Subtypes		
	Total Cohort (n = 110)	CM-1 (n = 66)	CM-1.5 (n = 44)	*p*
**Surgical findings**				
Arachnoid scarring				
- No/mild (scores = 0 or 1)	75 (68.1%)	50 (75.8%)	25 (56.8%)	**0.037 ^†^**
- Moderate/severe (scores = 2–4)	35 (31.9%)	16 (24.2%)	19 (43.2%)	**0.037 ^†^**
Tonsil coagulation or subpial resection				
- Yes	52 (47.3%)	18 (27.3%)	34 (77.3%)	**0.001 ^†^**
**Postoperative adverse events (PAE)**				
Patients without no PAE	77 (70%)	47 (71.2%)	30 (68.2%)	0.734
Patients with one or more PAE	33 (30%)	19 (28.8%)	14 (31.8%)	0.734
Adverse events				
- Fever syndrome related to PFR	25 (22.7%)	15 (22.7%)	10 (22.7%)	1.000
- CSF leaks	5 (4.5%)	2 (3%)	3 (6.8%)	
- Hydrocephalus	6 (5.5%)	3 (4.5%)	3 (6.8%)	
- Wound infection	1 (0.9%)	1 (1.5%)	-	
- Meningitis	1 (0.9%)	-	1 (2.3%)	
- Others	4 (3.6%)	3 (4.5%)	1 (2.3%)	
Effectiveness of surgery (surgical outcome, time of follow-up in years) *				
- Very good (6.5, 0.8 to 17.5)	101 (91.9%)	61 (92.4%)	40 (91%)	0.776
- Good (12.7, 0.7 to 15.7)	5 (4.5%)	3 (4.5%)	2 (4.5%)	
- Bad (12.2, 8.9 to 14.5)	4 (3.6%)	2 (3.1%)	2 4.5%)	

CM-1: CM type 1; CM-1.5: CM type 1.5. **^†^** Statistically significant differences between different groups of CM-1 vs. CM-1.5, *p* ≤ 0.05. * Median, minimum, and maximum.

## Data Availability

This study was conducted according to the FAIR principles committed to making data and services findable, accessible, interoperable, and reusable. The entire anonymized dataset, metadata, and dictionary are available and can be downloaded from Sahuquillo, J. (2024). Surgical Outcomes in Chiari 1 and Chiari 1.5 Malformation (pediatric cohort) [Data set]. Zenodo. https://doi.org/10.5281/zenodo.12166149 (accessed on 18 June 2024).

## Supplementary Materials

The following supporting information can be downloaded at: https://www.mdpi.com/article/10.3390/jcm13133852/s1. Figure S1. Additional information on the surgical technique; Video A-S1-Opening the FM.mp4; Video B-S1-Cerebellum_tonsils_arachnoid_membrane.mp4; Video E-S1-Dural graft closure.mp4; Video F-S1-Sealant on the dural graft.mp4; Case reports and lessons learned; Figure S2. Patient 2. First part; Figure S3. Patient 2. Second part; Figure S4. Patient 3. First part; Figure S5. Patient 3. Second part; Figure S6: (A) Control magnetic resonance imaging after implantation of a syringoperitoneal shunt, showing a reduction in the diameter of the Syr (2018). A year later (2019), the cavity had increased in size again, including the bulb. Note the patient’s opisthotonus position (B); Figure S7. Figure illustrating cerebellar slump in an adult patient after suboccipital craniectomy.

## Abbreviations

**AE:** adverse event; **AHI:** apnea/hypopnea index; **AMS:** aseptic meningitis syndrome; **AP:** anteroposterior **BI:** Basilar invagination; **CM-0:** Chiari malformation type 0; **CM-1:** Chiari malformation type 1; **CM-1.5:** Chiari malformation type 1.5; **CMs**: Chiari malformations; **CSF:** cerebrospinal fluid; **CVJ**: craniovertebral junction; **CXA:** clivoaxial angle; **EI:** Evans index; **FM:** foramen magnum; **ICC-CM:** International Consensus Conference on Chiari malformation; **ICD:** International Consensus Document; **ICP:** intracranial pressure; **MP-RAGE:** 3D magnetization prepared rapid gradient echo; **McRL:** McRae’s line; **MCAP:** megalencephaly–capillary malformation–polymicrogyria syndrome; **MeSHs:** medical subject headings; **MPR:** multiplanar reconstruction; **MRI:** magnetic resonance imaging; **PF:** posterior fossa; **PFD**: posterior fossa decompression; **PFR**: posterior fossa reconstruction; **PROSAC**: **PROS** pective registry of patients with Chiari malformations in Adults and Children; **SCI:** syrinx–canal index; **SCR:** syringo-cord ratio; **SG:** specific gravity; **Syr:** syringomyelia; **TH:** tonsillar herniation; **VHUH:** Vall d’Hebron University Hospital.
