# Supplementary material for: Surgical Outcomes in Chiari 1 and Chiari 1.5 Malformation Treated by Posterior Fossa Reconstruction: A Comprehensive Analysis of 110 Pediatric Cases and Literature Review
Source: J Clin Med. 2024 Jun 30;13(13):3852. doi: 10.3390/jcm13133852 (PMC11242314; doi:10.3390/jcm13133852)
Supplement: Supplementary file 1 [file jcm-13-03852-s001.zip › Supplementary Material_Surgery in pediatric Chiari malformations.pdf]

Supplementary Material

Additional information on the surgical technique

Figure S1 shows additional information on the surgical technique, with small pieces of videos trying to emphasize specific points.

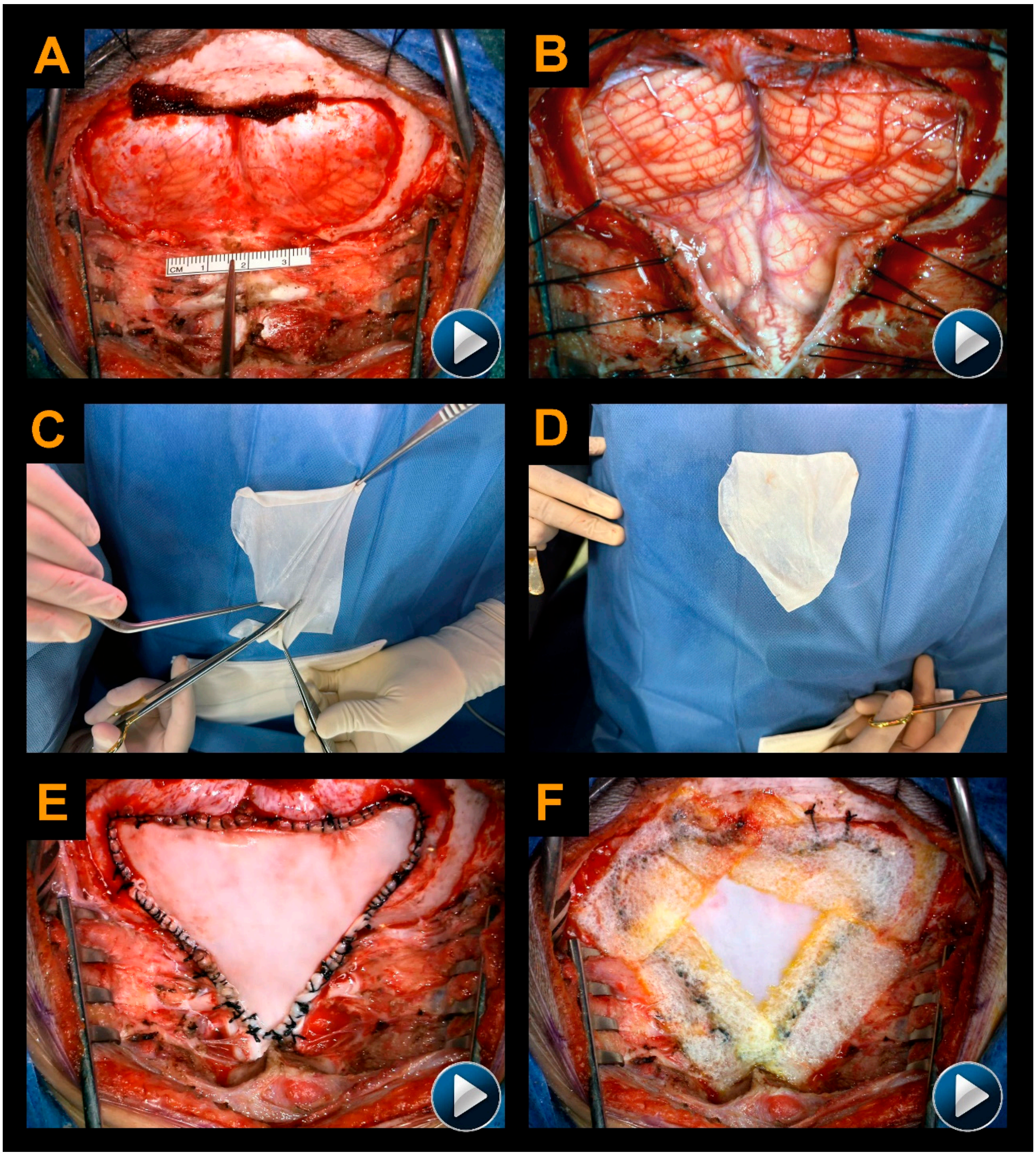

Figure S1: **A**. Dimensions of the suboccipital craniectomy (Video A-S1). **B**. Complete exposure of the cerebellum and tonsils with the arachnoid preserved intact; Video B-S1 shows the pulsatility of the neural structures. **C-D**. Preparation of the dural graft (bovine lyophilized pericardium allograft; Tutopatch®, BIO IMPLANTS Medical S.L, Barcelona, Spain). **E**. Watertight suture of the dural graft. Video E-S1 shows the level of CSF that forms under the dural graft and how the cardiac pulse is transmitted. **F**. Completed dural

closure with a sponge sealant patch (Tachosil®, Takeda Austria, Linz, Austria) reinforcing the suture line to avoid a CSF fistula. The sealant is placed such that there is approximately 1 cm on each side of the suture (Video F-S1)

Case reports and lessons learned

Patient 2. A four-year-old girl presented with cervical torticollis and scoliosis with a cervicothoracic scoliotic curve to the left. The neurological examination was normal. She underwent cranial and spinal magnetic resonance imaging (MRI) that revealed the presence of a Chiari malformation type 1.5 (CM-1.5) with a syringomyelic cavity that extended from C4 to D12 (**Figure S2**). The ventricular system was of normal size (EI= 0.28). In January 2009, she underwent posterior fossa reconstruction (PFR). The arachnoid was preserved intact without manipulation of the tonsils. The control MRI revealed a remodeling of the cerebellum with a small volume neocisterna magna and a modest reduction in the syringomyelic cavity in the thoracic region after 11 months and an improvement in the scoliotic curvature (**Figure S2**).

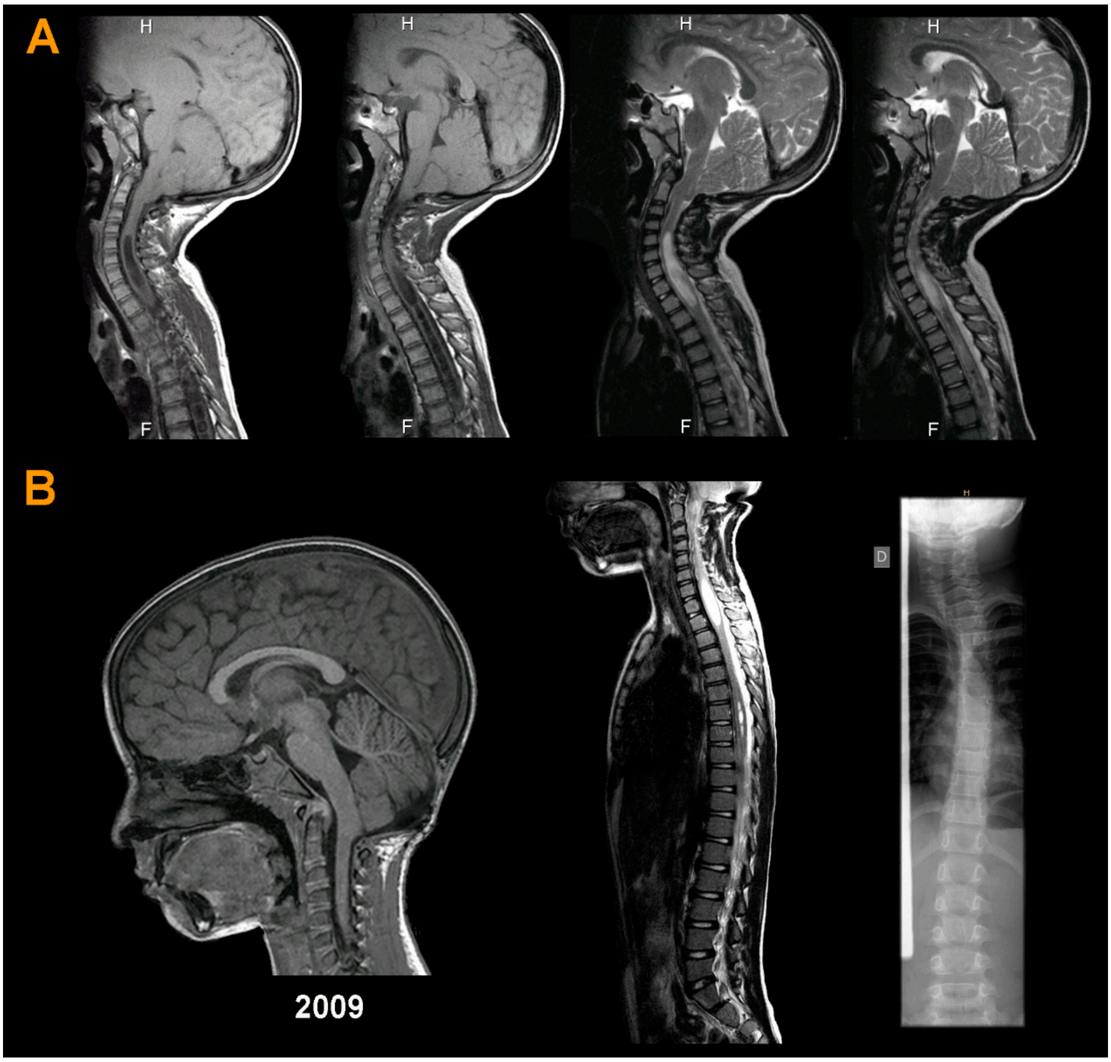

Figure S2: **A**. Magnetic resonance imaging (MRI) at diagnosis in 2008: T1-weighted on the left and T2-weighted on the right. **B**. MRI 11 months after posterior fossa reconstruction: cerebellar remodeling with a small cisterna magna on the left, and a reduction in the diameter of the syrinx cavity (center MRI) and of the scoliotic curve (right).

A new MRI two years after PFR showed a significant reduction in the volume of the cisterna magna and an increase in the Syr (**Figure S3**). The family was hesitant to consider new surgery until 2016, when, due to an increase in the Syr (**Figure S3**) and a new increase in scoliosis with worsening of SEP of the lower extremities, they agreed on a second surgery, which was conducted in 2017 at the age of 12. During surgery, new bone formation was observed over the dural graft with severe arachnoiditis and scarring between the graft and the cerebellum. After removing the previous dural graft, we used a synthetic GORE® dural graft (W. L. Gore & Associates, Inc., Phoenix, AZ,

USA) with the intention of promoting the local accumulation of cerebrospinal fluid (CSF) to prevent new adhesions between the muscular layer and the cerebellum. The outcome was good, the Syr was reduced significantly and the scoliotic curve improved (**Figure S3**). These changes have persisted over time and the girl has resumed a normal life.

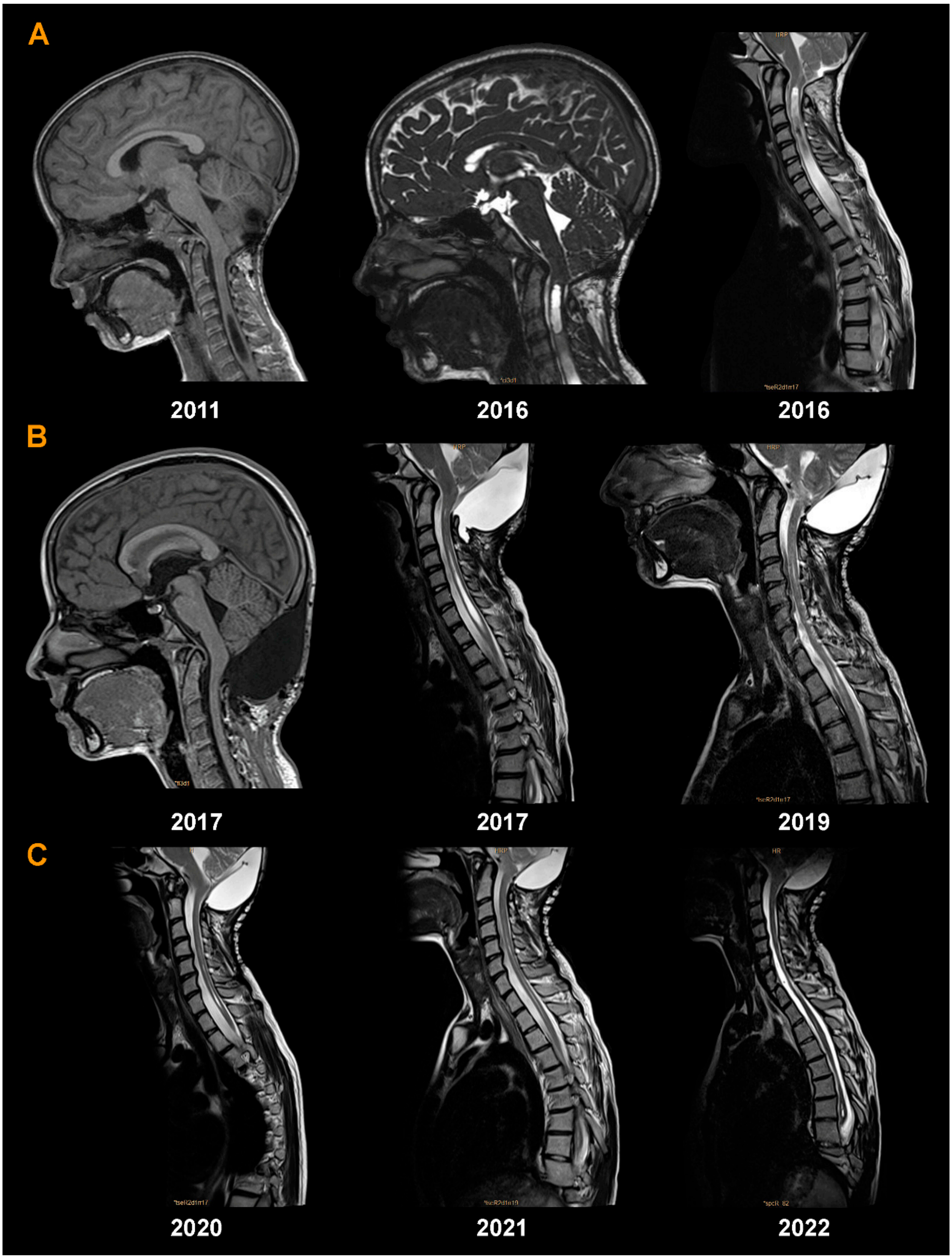

Figure S3: **A**. Control magnetic resonance imaging (MRI) in 2011 showed a volumetrically reduced artificial cisterna magna. The MRI study conducted in 2016 showed an increase in the extension and diameter of the Syr. **B**. Control MRI performed one year after the second surgery on the posterior fossa (2017) with a pseudo meningocele and a marked reduction of the syrinx size; these changes were even more evident in the 2019 MRI. **C**. The anatomical improvement of the second surgery persisted in the successive MRIs of 2020-2022.

Based on the scores presented in the article, the patient's one-year post-surgery outcome could be considered good (clinical outcome= very good, because of the improvement in the scoliotic curve; neuroimaging outcome= good, due to the changes on the PF but only a moderate reduction of the Syr; surgical outcome= good). This patient underscores how important continuous surveillance is for children who still have Syr and how the simple apposition of a GORE® dural graft constitutes a good option for rescue surgeries as it avoids scarring between the muscle layers and the neural tissue. However, this type of graft is not recommended at first surgery.

Patient 3: In 2008, a five-year-old immigrant boy was taken to the emergency room due to headaches and vomiting. Neuroimaging revealed CM-1, severe hydrocephalus, and an extensive Syr (C2-D12) (**Figure S4**). The patient's examination on admission showed a significant increase in head circumference (macrocephaly) and a delay in his psychomotor development. The patient was implanted with a ventriculoperitoneal CSF shunt (Miethke Paedi GAV® 9/19, Aesculap, AG, Tuttlingen, Germany). This resulted in a significant reduction in ventricular size and the disappearance of headaches and vomiting. Patient follow-up was difficult due to language barriers and repeated absences from scheduled check-ups. Four years after surgery (2012), the patient was referred again to the Pediatric Neurosurgery outpatient clinic because of headaches and swallowing difficulties. The control MRI confirmed that the shunt function was correct (EI=0.29) (**Figure S4**). However, given the persistence of the extensive Syr, the patient underwent a direct approach to the CM-1. During surgery, it was observed that the arachnoid re-expanded well, there was no arachnoiditis, and the integrity of the arachnoid was preserved.

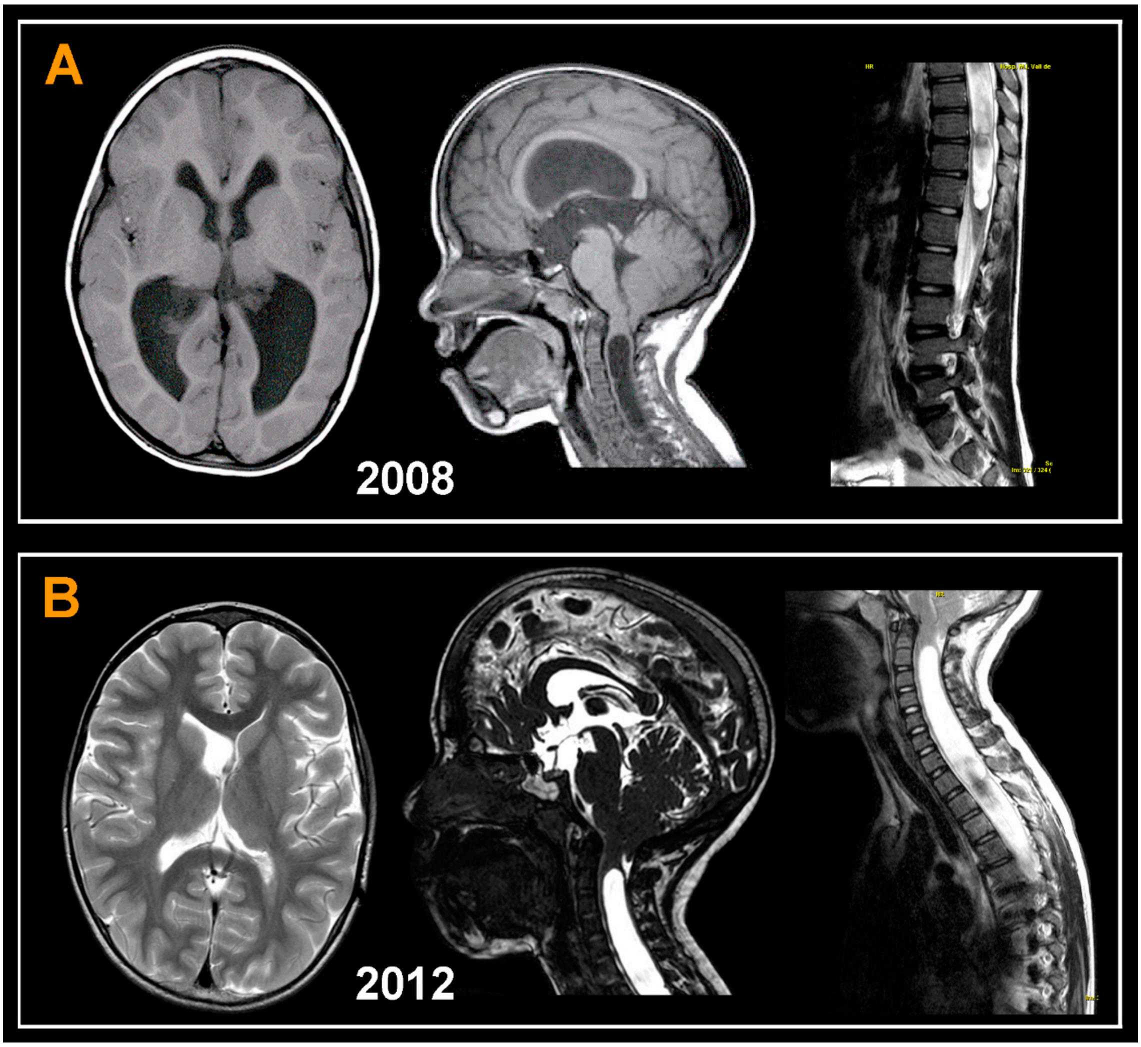

Figure S4: **A**. Magnetic resonance imaging (MRI) performed in 2008 during the first admission of the patient, in whom significant ventriculomegaly was observed, in the context of a Chiari malformation type 1 and extensive holomedullary syringomyelia. **B**. A control MRI four years later detected a reduction in the size of the ventricular system without any relevant changes in either the CM-1 or the Syr.

The patient did not present any post-surgical adverse event and his symptoms improved, with the presence in the control MRI of a small neocisterna magna and a reduction in the diameter of the Syr (**Figure S5**). However, the scoliotic curve progressed and required surgery in 2015 (T2-L4). Despite these surgical procedures, Syr continued progressing with important neurological worsening and motor and sensory deficits in all four extremities (**Figure S5**).

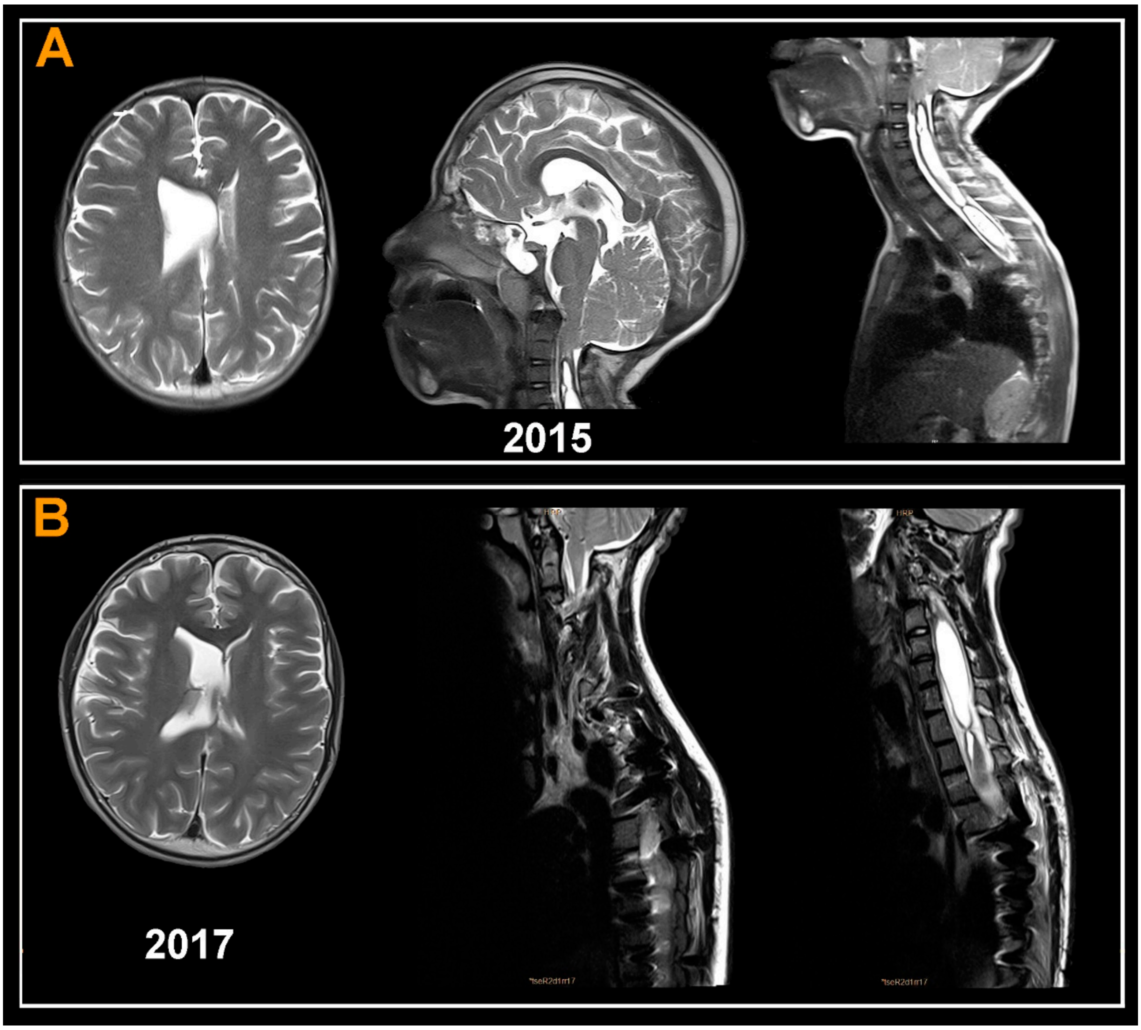

Figure S5: **A**. Control magnetic resonance imaging after posterior fossa reconstruction shows the persistence of a small neocisterna magna and the reduction in the diameter of the syringomyelic cavity (2015). The patient's neuroimaging remained stable until 2017 when an increase in the diameter of the Syr was observed (**B**).

Because of this neurological worsening, we decided to place a syringoperitoneal shunt (2018), which was effective in reducing the diameter of the Syr (**Figure S6**). In the following months, however, the Syr continued to progress, as did the patient's neurological deterioration, with lower cranial nerve involvement that required a tracheostomy (**Figure S6**). We decided to perform a second surgery to rescue the syringobulbia. During this last surgery (2019), the previous dural graft had severe scarring and adhesions to the cerebellum. The graft was removed and a myelotomy was performed in the DREZ region of C1 with the introduction of a GORE® stent. Also, a new GORE® dural graft was placed without suturing. Despite all these surgical maneuvers, the patient's situation did not improve, presenting severe and progressive spasticity. In

this context, the family rejected other therapeutic maneuvers, and the patient died in 2019 at the age of 16.

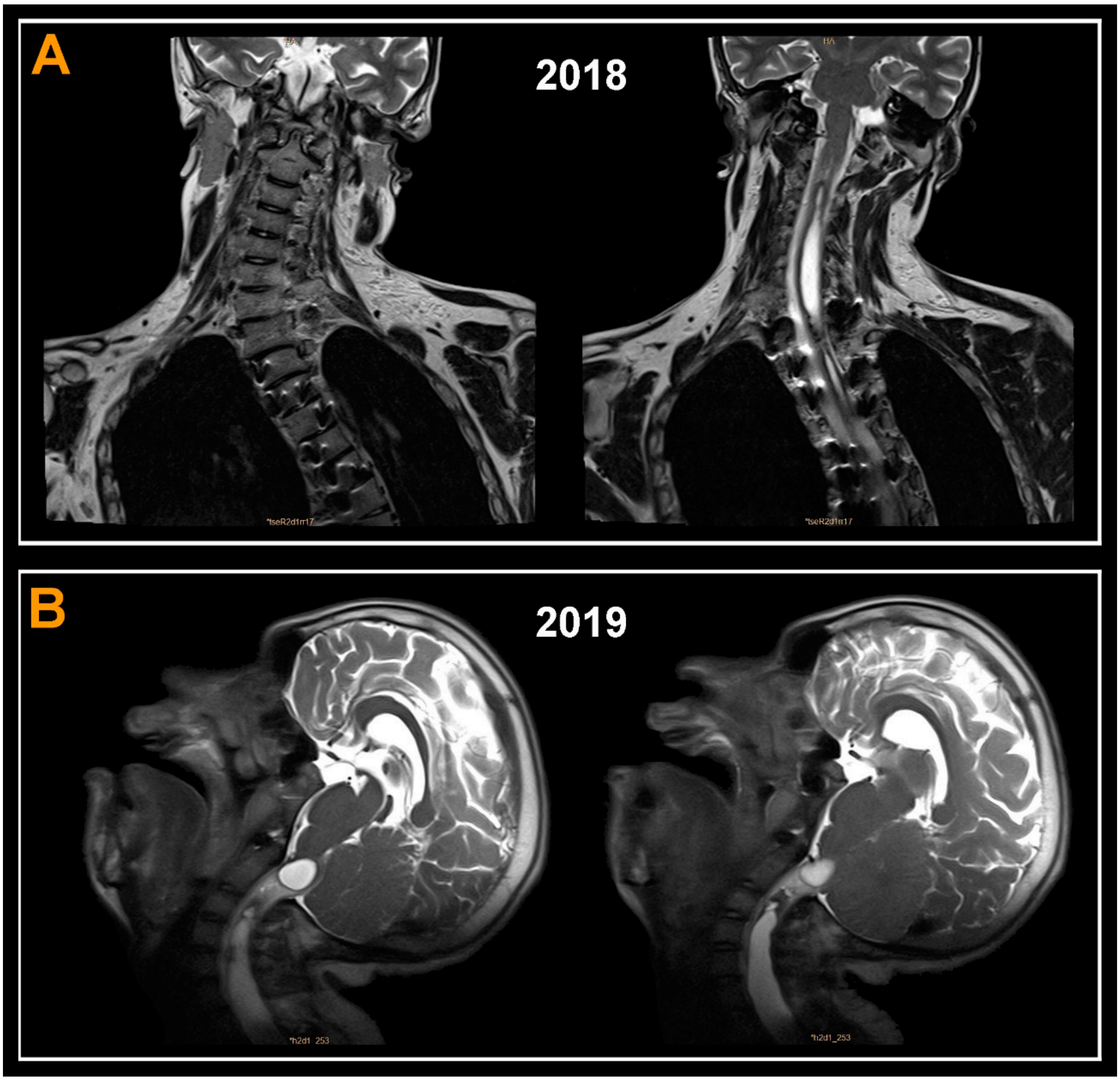

Figure S6: **A**. Control magnetic resonance imaging after implantation of a syringoperitoneal shunt, showing a reduction in the diameter of the Syr (2018). A year later (2019), the cavity had increased in size again, including the bulb. Note the patient's opisthotonus position (**B**)

Considerations about the case. In light of this patient's poor neurological development, we believe the following topics should be emphasized: **1.** When CM-1 and a Syr present together with hydrocephalus, early treatment with a shunt is encouraged; in this particular case, it might have avoided the patient's psychomotor delay. **2.** After treating hydrocephalus, the direct CM-1 approach should have been addressed to manage Syr; **3.** Clinical changes in patients do not necessarily correspond with changes in Syr size and extension. In this child, the neurological worsening continued even after the Syr's

diameter decreased. In summary, this particular case underscores the importance of providing these patients with timely and adequate care to prevent initiating treatment at an advanced stage, when it is frequently impossible to interrupt the disease's progression.

Figure illustrating cerebellar slump in an adult patient after suboccipital craniectomy

For a better understanding of the significance of suboccipital craniectomy size, please see the discussion section (4.2 Decompressing vs. Reconstructing the Posterior Fossa).

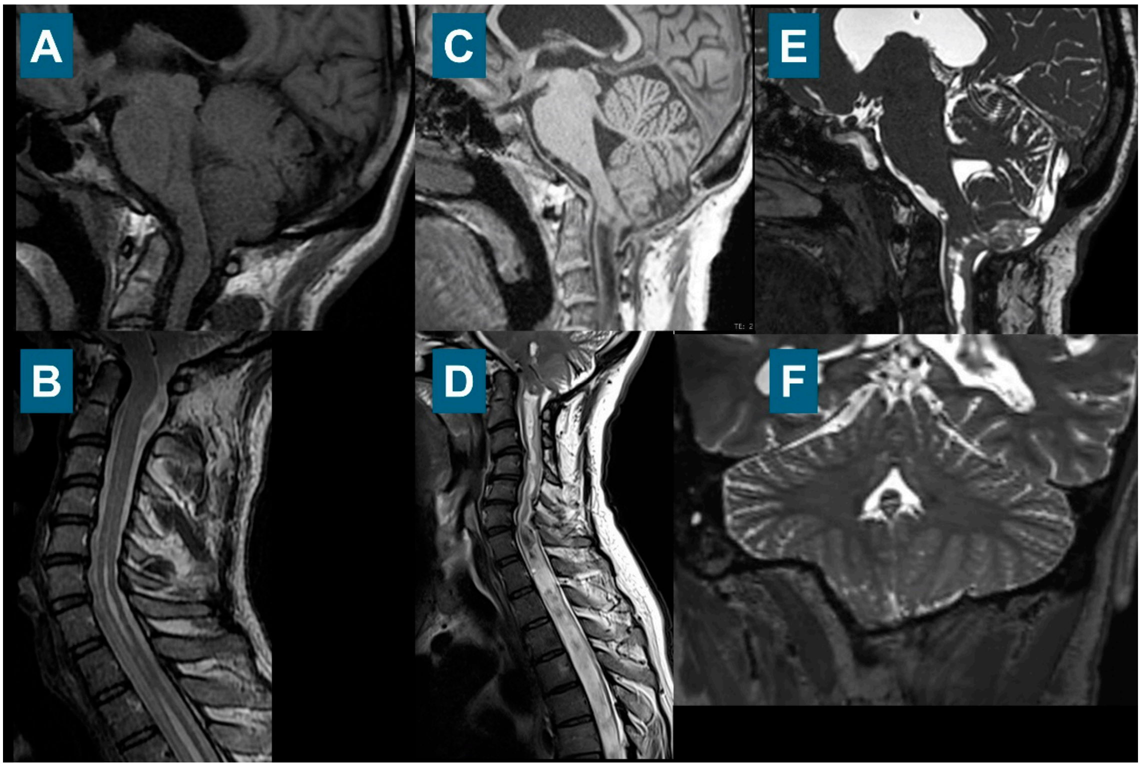

Figure S7. An example of a typical cerebellar ptosis or 'slump' into the posterior fossa (PF) craniectomy in a 50-year-old male operated on for CM-1.5 because of headaches and unstable gait. **A.** Sagittal magnetic resonance imaging (MRI) showing a crowded PF and tonsillar herniation with the tip of the tonsils reaching the level of the posterior arch of C1. **B.** T2-weighted sagittal MRI showing a cervico-thoracic Syr and hydrocephalus. The patient underwent a posterior fossa decompression with opening the dura mater and a bovine-collagen dural graft at another institution. The patient developed a CSF fistula that was solved with lumbar evacuation of the CSF and compressive dressings. After discharge, the patient showed a slow, progressive neurological deterioration with decreased hand grip and slowed gait. Over a 24-month period, he reported right upper extremity dysesthesia, progressive tetraparesis, and loss of bladder control. **C.** Sagittal T1-T2-weighted MRI two years after surgery showed cerebellar ptosis into a large suboccipital craniectomy but without the formation of a CSF cushion below the

cerebellum. **D**. T2-weighted sagittal MRI showing a significant progression of the Syr, extending from the medulla to the thoracic spinal cord, which was the cause of the progressive neurological worsening. **E.** Sagittal T2-weighted MRI at the same level as C. **F.** Coronal T2-weighted slice showing the herniated biventral lobes and the complete absence of a CSF cushion below the cerebellum that could restore its neutral buoyancy. The patient underwent a ventriculoperitoneal shunt and, six months later, a syringe-pleural shunt as a rescue therapy without any significant improvement afterward.
